# Biomimetic Strategies for Nutraceutical Delivery: Advances in Bionanomedicine for Enhanced Nutritional Health

**DOI:** 10.3390/biomimetics10070426

**Published:** 2025-07-01

**Authors:** Vicente Javier Clemente-Suárez, Alvaro Bustamante-Sanchez, Alejandro Rubio-Zarapuz, Alexandra Martín-Rodríguez, José Francisco Tornero-Aguilera, Ana Isabel Beltrán-Velasco

**Affiliations:** 1 Faculty of Medicine, Health and Sports, Universidad Europea de Madrid, Villaviciosa de Odón, 28670 Madrid, Spain; vctxente@yahoo.es (V.J.C.-S.); alvaro.bustamante@universidadeuropea.es (A.B.-S.); 2 Grupo de Investigación en Cultura, Educación y Sociedad, Universidad de La Costa, Barranquilla 080002, Colombia; 3 Faculty of Health Sciences, UNIE, 28015 Madrid, Spain; 4 Graduate School of Business, Universidad ESAN, Alonso de Molina 1652, Santiago de Surco, Lima 15023, Peru; 5 Psychology Department, Facultad de Ciencias de la Vida y la Naturaleza, Universidad Antonio de Nebrija, 28240 Madrid, Spain; abeltranv@nebrija.es

**Keywords:** biomimetic delivery, nutraceuticals, bioavailability enhancement, antioxidant nanocarriers, biogenic nanoparticles, targeted nutrient delivery

## Abstract

Background: Biomimetic strategies have gained increasing attention for their ability to enhance the delivery, stability, and functionality of nutraceuticals by emulating natural biological systems. However, the literature remains fragmented, often focusing on isolated technologies without integrating regulatory, predictive, or translational perspectives. Objective: This review aims to provide a comprehensive and multidisciplinary synthesis of biomimetic and bio-inspired nanocarrier strategies for nutraceutical delivery, while identifying critical gaps in standardization, scalability, and clinical translation. Results: We present a structured classification matrix that maps biomimetic delivery systems by material type, target site, and bioactive compound class. In addition, we analyze predictive design tools (e.g., PBPK modeling and AI-based formulation), regulatory frameworks (e.g., EFSA, FDA, and GSRS), and risk-driven strategies as underexplored levers to accelerate innovation. The review also integrates ethical and environmental considerations, and highlights emerging trends such as multifunctional hybrid systems and green synthesis routes. Conclusions: By bridging scientific, technological, and regulatory domains, this review offers a novel conceptual and translational roadmap to guide the next generation of biomimetic nutraceutical delivery systems. It addresses key bottlenecks and proposes integrative strategies to enhance design precision, safety, and scalability.

## 1. Introduction

Biomimicry, defined as the emulation of models and systems from nature to solve complex human challenges, has become an influential paradigm in the development of advanced biomedical and nutraceutical technologies. In the context of nutritional science, biomimetic strategies have led to the design of smart delivery systems that imitate cellular membranes, metabolic pathways, and intercellular transport mechanisms to enhance the performance of bioactive compounds. These systems are particularly valuable in overcoming limitations commonly associated with conventional nutraceuticals, such as instability in gastrointestinal environments, low water solubility, and poor systemic bioavailability [1]. Recent breakthroughs in nanotechnology have enabled the fabrication of biomimetic carriers, including liposomes, polymeric nanoparticles, and nanoemulsions, that protect sensitive compounds from premature degradation, control their release profiles, and facilitate targeted delivery to specific tissues [2]. Furthermore, cell membrane-coated nanocarriers and hybrid systems derived from natural biomaterials are increasingly recognized for their capacity to evade immune detection and interact more effectively with biological barriers, thereby expanding the therapeutic horizon of nutraceutical interventions [3,4]. As chronic diseases and oxidative stress-related disorders rise globally, the integration of bio-inspired design into nutritional therapies is no longer a futuristic proposition but a present-day necessity, offering new possibilities for safe, efficient, and precise nutrient delivery [5,6].

The global nutraceuticals market was valued at over USD 450 billion in 2023 and is projected to exceed USD 700 billion by 2030, driven by rising consumer demand for health-promoting, disease-preventive functional products. Within this space, nano-nutraceuticals—formulations enhanced through nanotechnology—are experiencing rapid growth due to their superior bioavailability and targeted delivery properties [7]. Recent forecasts suggest the nano-nutraceutical sector will grow at a compound annual growth rate (CAGR) of over 9%, particularly in domains such as antioxidants, omega-3 fatty acids, and herbal extracts. These trends underscore the pressing need for innovative and safe delivery strategies, such as biomimetic nanocarriers, to meet both efficacy and regulatory demands in a highly dynamic market [7,8].

A central factor driving the interest in biomimetic nutraceutical systems is their potential to mitigate oxidative stress—an imbalance between the generation of reactive oxygen species (ROS) and the body’s ability to neutralize them through endogenous antioxidants. Oxidative stress is a key pathological mechanism implicated in the development and progression of numerous chronic diseases, including cardiovascular disorders, neurodegenerative conditions, and metabolic syndromes [9]. Nutraceuticals rich in antioxidants, such as polyphenols, flavonoids, and carotenoids, offer promising therapeutic effects but suffer from intrinsic limitations like instability in physiological environments and limited bioefficacy upon oral administration [10,11]. Recent advances in biomimetic nanocarrier design, including cell membrane-coated nanoparticles, micelle-based systems, and hybrid biomaterials, have enabled more effective delivery of such antioxidants, improving their solubility, permeability, and targeted cellular uptake [3,12]. For instance, biomimetic nanoparticles encapsulating *Prunus spinosa* extract demonstrated enhanced anti-inflammatory and antioxidant activity in vitro, validating their potential for wound healing and oxidative damage control [13]. Additionally, selenium-based nanostructures functionalized with natural ligands have been engineered to cross biological barriers and deliver redox-active compounds directly to neural tissues, highlighting their applicability in neuroprotective therapies [14]. These innovations mark a significant departure from conventional supplement formulations, positioning biomimetic platforms as pivotal tools in the next generation of nutritional therapeutics focused on redox homeostasis.

A major obstacle in the clinical translation of nutraceuticals is their inherently low oral bioavailability, which significantly limits their therapeutic potential. Factors such as poor aqueous solubility, degradation in the gastrointestinal tract, and limited permeability across epithelial barriers hinder the effective absorption and systemic distribution of many bioactive compounds [15]. Biomimetic delivery systems have emerged as a strategic solution to this challenge by mimicking biological membranes and leveraging endogenous transport mechanisms to enhance bio accessibility and absorption. For example, protein-based nanocarriers and micellar structures have demonstrated superior performance in improving the pharmacokinetic profiles of hydrophobic nutrients such as lutein, curcumin, and astaxanthin, which are otherwise poorly absorbed [16,17]. A recent study reported that macrophage membrane-coated nanoparticles loaded with lutein not only improved intestinal uptake but also exhibited targeted therapeutic effects in cardiac tissue, highlighting their dual role in bioavailability enhancement and tissue-specific delivery [16]. Similarly, biocompatible selenium nanoparticles engineered with functional ligands and lipid coatings have shown promising results in increasing nutrient retention in neural tissues, with significant implications for treating neurodegenerative conditions [14]. These findings underscore the growing role of biomimetic nanoformulations as effective vectors for optimizing nutraceutical efficacy, enabling precision nutrition strategies tailored to individual physiological needs.

Recent developments in microbiota-based biomimetics further illustrate the expanding frontiers of bio-inspired technologies in health and nutritional science. The human gut microbiota—recognized as a complex and resilient ecosystem—has emerged not only as a critical modulator of host physiology but also as a conceptual blueprint for designing adaptive and self-regulating delivery systems [18]. The intricate functions of microbial communities, including quorum sensing, ecological adaptation, and metabolite production, have inspired the engineering of nano-scale systems capable of interacting intelligently with host environments. These include microbiota-mimicking biosensors, membrane-coated vesicles, and metabolite-responsive drug delivery platforms that mirror microbial communication and metabolic responsiveness. Such innovations underscore the relevance of integrating microbiome principles into nutraceutical formulation, particularly for addressing challenges related to intestinal homeostasis, systemic inflammation, and metabolic dysregulation [14,19]. The rise in precision therapies based on artificial microbiomes and synbiotic nanostructures reflects a broader trend in which biomimetic design is converging with personalized nutrition, allowing for tailored interventions that emulate the dynamic equilibrium observed in healthy microbial ecosystems. This conceptual alignment between microbiota resilience and advanced delivery mechanisms offers a valuable perspective from which to explore the next generation of nutraceutical platforms.

Despite the growing body of research highlighting the advantages of biomimetic systems in nutraceutical delivery, the field remains fragmented, with limited integration of findings across different application domains. Studies often focus on isolated outcomes—such as antioxidant performance, bioavailability enhancement, or anti-inflammatory effects—without establishing a comprehensive understanding of how these mechanisms interact within the broader context of nutritional health and chronic disease management [20,21]. Moreover, regulatory challenges and safety considerations remain underexplored, particularly concerning the long-term biocompatibility and potential toxicity of novel biomimetic nanomaterials. Given the accelerating prevalence of non-communicable diseases globally, there is a pressing need for an updated, multidisciplinary synthesis of the current advancements in bio-inspired delivery systems that can address these multifaceted challenges. Given the growing complexity and diversity of biomimetic delivery systems applied to nutraceuticals, there is a critical need for an integrative review that synthesizes recent evidence and evaluates translational potential across multiple health domains. Although numerous studies have demonstrated enhanced antioxidants and anti-inflammatory outcomes using biomimetic formulations, the field lacks a cohesive framework that connects these molecular effects with long-term clinical implications in chronic disease prevention [10,19]. Furthermore, emerging strategies, including the use of membrane-coated vesicles, biopolymeric scaffolds, and stimuli-responsive nanocarriers, have shown significant promise but remain underreported in the context of nutraceutical science. To address this gap, the present review critically examines the current state of the art in biomimetic and biogenic nanoparticle applications for nutraceutical delivery. The objectives are three-fold: (1) to assess recent technological innovations that enhance the bioavailability and therapeutic efficacy of nutritional compounds, (2) to evaluate the role of these systems in managing oxidative stress and inflammation, and (3) to highlight their emerging relevance in the prevention and modulation of chronic diseases. This comprehensive synthesis aims to offer researchers and clinicians a consolidated reference point for guiding future investigations and advancing personalized nutritional interventions through bionanotechnology.

To ensure conceptual clarity and avoid terminological ambiguity, a glossary of key terms used throughout this review is presented in Table 1. Given the increasing overlap between fields such as nanomedicine, biomaterials, and nutraceuticals, the distinction between terms like biogenic, bio-inspired, biomimetic, and nanozymes is essential for accurately interpreting the scope and implications of each strategy. This standardized terminology aims to support consistent understanding among multidisciplinary readers.

## 2. Methodology of Literature Selection

The protocol for this review adhered to a structured and systematic literature search strategy formulated to ensure the rigorous identification, selection, and evaluation of scientific literature relevant to the field of biomimetic and bio-inspired delivery systems for nutraceutical applications. The methodological design of the search process was rooted in established guidelines for evidence synthesis in biomedical and nutritional sciences, with an emphasis on reproducibility, transparency, and comprehensiveness. To ensure robustness and breadth, the search integrated both primary sources—namely, peer-reviewed original research articles and review papers published in scientific journals—and secondary sources, including authoritative bibliographic repositories and scholarly indexes. These included PubMed, Scopus, Web of Science, ScienceDirect, Embase, and SpringerLink, all of which are globally recognized databases with extensive coverage of biomedical, pharmaceutical, and materials science literature. Each database was explored independently to minimize publication bias and to capture a wide spectrum of studies across different scientific disciplines that intersect within the domain of bionanotechnology and nutritional health.

A search syntax was constructed for each database, making use of Boolean operators (AND, OR, and NOT) and controlled vocabularies such as Medical Subject Headings (MeSH) in PubMed and Emtree terms in Embase where applicable. This approach facilitated both sensitivity and specificity in retrieving relevant records. For example, combined terms such as “(biomimetic OR bio-inspired OR nature-inspired) AND (nanoparticles OR nanocarriers OR nanoemulsions) AND (nutraceutical OR dietary supplement OR functional food) AND (delivery OR encapsulation OR bioavailability)” were adapted and refined iteratively for each database interface. Truncations, wildcards, and proximity operators were also used where supported to expand the reach of the search and capture semantic variations in the literature. The protocol also included citation tracking of key papers, snowball sampling from bibliographies, and examination of recent special issues in high-impact journals related to nanomedicine, pharmaceutical sciences, food technology, and biomedical engineering. This triangulated strategy was intended to encompass both emerging experimental evidence and comprehensive reviews that provide theoretical and mechanistic insights into biomimetic strategies. The search process was documented and saved for reproducibility and future updates of this review.

The search was limited to publications from January 2015 to March 2025, with priority given to articles published in the last five years. Only peer-reviewed journal articles indexed in JCR (Journal Citation Reports) or SJR (Scimago Journal Rank) were considered eligible. The keywords and search phrases used included a combination of general and specific terms, such as *biomimetic nanoparticles*, *bio-inspired nanocarriers*, *biogenic delivery systems*, *nanotechnology in nutraceuticals*, *antioxidant nanocarriers*, *anti-inflammatory nanoformulations*, *nutraceutical bioavailability*, *targeted nutrient delivery*, *bionanomedicine*, *nutritional nanotechnology*, *oxidative stress modulation*, *chronic disease prevention with nutraceuticals*, *membrane-coated nanoparticles*, *natural polymer-based nanocarriers*, *curcumin delivery systems*, *selenium nanoparticles*, *cell-membrane mimicry*, and *nutritional health and nanomedicine*.

Articles were included if they (i) reported original experimental or review data on biomimetic or biogenic nanoparticle applications in nutraceutical delivery; (ii) addressed outcomes related to bioavailability, antioxidant activity, anti-inflammatory efficacy, or chronic disease modulation; and (iii) were published in high-impact journals in the fields of nanomedicine, food science, pharmacology, or biomedical engineering. Foundational works published before 2015 were retained only when they provided essential mechanistic or theoretical insights, aligned with established procedures [22,23,24,25,26,27].

Studies were excluded if they (i) presented obsolete findings no longer consistent with current nanobiotechnological standards; (ii) focused on unrelated biomedical technologies or pharmaceutical drug delivery without nutritional context; or (iii) were dissertations, editorials, abstracts, conference proceedings, books, or unpublished materials not subject to peer review. All the selected articles were assessed for methodological quality, relevance, and publication in indexed sources to ensure scientific robustness and credibility.

**Figure 1:**
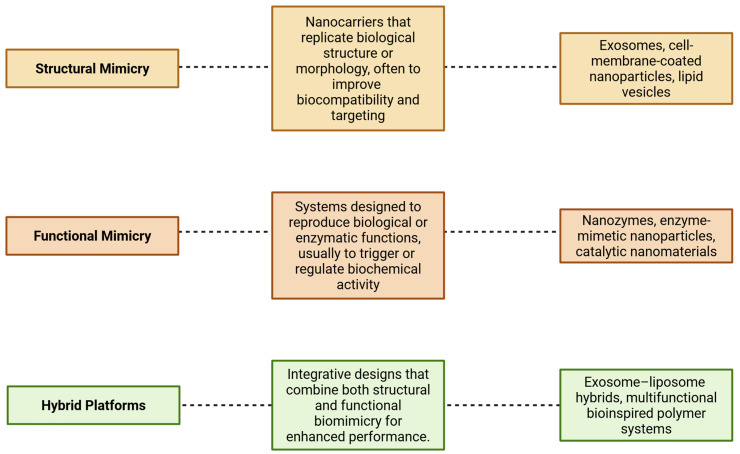
Conceptual framework for biomimetic systems.

To provide a conceptual basis for understanding the systems discussed in this review, we propose a functional framework organized around three biomimetic design categories (Figure 1): (1) structural mimicry—systems that emulate biological architecture, such as cell-derived vesicles or exosomes; (2) functional mimicry—systems that replicate enzymatic or molecular functions, such as nanozymes; and (3) hybrid platforms—integrated designs that combine both aspects, such as engineered exosome–liposome systems. This classification supports a cohesive understanding of the technological continuum within biomimetic nanocarrier development.

## 3. Foundations of Biomimetic Nanotechnology

The growing interest in biomimetic approaches for nutraceutical delivery reflects a broader shift toward functional and targeted nutritional strategies that mimic biological processes for enhanced therapeutic efficacy. On this line, conventional delivery systems often fail to address the multifaceted challenges associated with nutraceuticals, including poor water solubility, chemical instability, low permeability, and rapid degradation within the gastrointestinal tract [28]. In contrast, biomimetic encapsulation systems engineered to replicate structures and behaviors observed in nature present a promising paradigm for stabilization, controlled release, and bioavailability enhancement of bioactive dietary compounds. Therefore, biomimetic encapsulation systems are inspired by the organization and functionality of natural biological assemblies such as cell membranes, lipoproteins, extracellular vesicles (e.g., exosomes), and protein-based nano compartments [29]. These systems are typically constructed from biocompatible materials such as phospholipids, natural proteins, polysaccharides, or hybrid combinations that emulate natural transport mechanisms, thereby facilitating greater physiological compatibility and enhanced interaction with biological membranes. Furthermore, these systems may exploit active targeting mechanisms by incorporating ligands, peptides, or membrane proteins that enable receptor-mediated endocytosis and selective tissue targeting [30].

At the core of biomimetic encapsulation lies the principle of *form–function congruence*: mimicking the molecular architecture and dynamic behavior of natural systems to achieve equivalent performance in a synthetic or semi-synthetic carrier. Key design criteria include structural stability, protection from digestive enzymes, resistance to pH variation, controlled-release capabilities, and the ability to cross biological barriers. One of the central strategies involves mimicking cellular phospholipid bilayers to form vesicular structures such as liposomes or niosomes [31]. These systems can encapsulate both hydrophilic and hydrophobic nutraceuticals, forming highly adaptable vehicles with established safety profiles. Another approach leverages natural vesicle systems, such as exosomes or milk-derived nanovesicles, which inherently possess targeting ligands and transport mechanisms evolved for intercellular communication [32]. In addition to vesicular systems, protein-based assemblies such as casein micelles, zein nanoparticles, or gelatin microcapsules offer structural plasticity and responsiveness to environmental triggers like pH, temperature, or enzymatic activity. These systems are particularly attractive for orally delivered compounds that must withstand gastric conditions and release selectively in the intestine [33]. Moreover, polysaccharide-based capsules, such as those made from chitosan, alginate, pectin, or gum Arabic, further extend the repertoire of biomimetic systems. These carriers exhibit mucoadhesive properties and can transiently open tight junctions in intestinal epithelium, thereby improving paracellular transport of encapsulated bioactives [34,35]. Importantly, they can be modified with surface charges or functional groups to optimize cellular interaction and uptake.

Liposomes are spherical vesicles composed of lipid bilayers that closely resemble biological membranes. Their amphiphilic nature enables them to encapsulate a diverse range of compounds, including curcumin, EGCG, resveratrol, and coenzyme Q10. Recent advances include stealth liposomes, which incorporate polyethylene glycol (PEG) or are coated with cellular membrane fragments to evade macrophage clearance and prolong systemic circulation [36]. Functionalization with targeting ligands such as folic acid or transferrin has further enhanced tissue specificity, particularly for intestinal and hepatic delivery. Exosomes and exosome-mimetic nanovesicles are gaining traction due to their natural origin, biocompatibility, and ability to mediate intercellular delivery. Engineered exosome-like structures can be derived via cell extrusion, sonication, or microfluidic assembly, and are used to encapsulate hydrophobic nutraceuticals such as vitamin D3 and omega-3 fatty acids. Their surface proteins and membrane composition facilitate active uptake via endocytosis and may enable the crossing of biological barriers such as the blood–brain barrier, opening new avenues for neuroprotective nutraceutical interventions [37]. Also, natural proteins such as casein, whey, gelatin, albumin, and plant-derived zein are increasingly used in biomimetic encapsulation due to their biodegradability and responsiveness to physiological cues. These proteins can self-assemble into nanoparticles or nano micelles under specific pH or ionic conditions, encapsulating lipophilic nutraceuticals while offering gastrointestinal protection. For instance, casein micelles have been shown to stabilize curcumin and enhance its intestinal absorption, while gelatin nanoparticles improve the gastric resistance of polyphenols and vitamins [38]. In addition, chitosan and alginate nanoparticles exhibit pH-sensitive swelling and mucoadhesive properties that enhance the residence time of nutraceuticals in the gastrointestinal tract. These systems can be tailored to release bioactives in the colon or small intestine and are frequently used in synbiotic delivery systems combining probiotics and prebiotics. Hybrid formulations that combine proteins and polysaccharides (e.g., gelatin–chitosan or zein–pectin) provide synergistic advantages in terms of encapsulation efficiency, particle stability, and bioavailability [39].

Furthermore, biomimetic encapsulation systems offer several functional advantages over conventional nutraceutical formulations, primarily due to their capacity to mimic natural biological interfaces and delivery mechanisms. These systems are designed not only to protect labile bioactive compounds from degradation but also to control their release, improve solubility, increase intestinal permeability, and enhance cellular uptake, all critical parameters for optimizing the physiological effects of nutraceuticals. One of the most compelling demonstrations of efficacy is seen in enhanced bioavailability. Numerous studies have reported that liposome-encapsulated nutraceuticals such as curcumin, coenzyme Q10, and lycopene exhibit significantly higher plasma concentrations than their unencapsulated counterparts. In animal models, liposomal curcumin has shown up to five-fold greater systemic bioavailability, attributed to the protection of the compound from rapid metabolism and improved absorption via passive diffusion or endocytosis mechanisms [40]. Similarly, self-assembled phospholipid vesicles have been shown to improve the oral absorption of poorly soluble antioxidants such as astaxanthin and quercetin [41]. In the context of exosome-like vesicles, several in vitro and in vivo studies have revealed that these carriers promote efficient cellular uptake via clathrin-mediated endocytosis and are preferentially internalized by target cells due to their surface ligands and membrane protein signatures. For example, exosome-mimetic nanovesicles encapsulating resveratrol have demonstrated significantly enhanced uptake in Caco-2 and HepG2 cell lines, resulting in improved intracellular antioxidant activity and reduced oxidative damage [42].

Further, protein-based encapsulation systems also contribute to enhanced functional efficacy by offering dynamic release behavior. Casein micelles, for example, disassemble in response to intestinal pH and enzymatic activity, releasing their cargo in a temporally controlled manner. This strategy not only improves the intestinal residence time of compounds like catechins and curcuminoids but also supports sustained antioxidant and anti-inflammatory effects in vivo. Zein nanoparticles, derived from maize protein, have shown efficacy in encapsulating lipophilic vitamins and polyphenols, exhibiting a slow-release pattern in simulated gastrointestinal fluid and improving total antioxidant capacity in rat plasma [43]. Hybrid carriers that integrate multiple biomimetic elements such as lipid–protein complexes or polysaccharide–lipid nanoparticles enable the co-delivery of synergistic compounds. One notable example is the co-encapsulation of curcumin and piperine within a protein–lipid hybrid nanoparticle, which led to improved pharmacokinetic parameters and greater suppression of inflammatory cytokines in murine models of colitis [44]. Another study demonstrated that quercetin–rutin combinations delivered via gelatin–alginate core–shell nanoparticles produced a synergistic reduction in ROS and lipid peroxidation in endothelial cells under oxidative stress [45]. A particularly important aspect of biomimetic systems is their ability to target delivery to specific tissues or cells, thus enhancing therapeutic specificity. For example, folic acid-conjugated liposomes have been used to deliver vitamin E and flavonoids selectively to inflamed intestinal tissue, while exosome-like vesicles derived from milk have demonstrated preferential accumulation in the colon, making them suitable for targeted delivery in inflammatory bowel disease (IBD) models [46]. Such targeting not only amplifies efficacy but also reduces systemic exposure and potential side effects.

Furthermore, some biomimetic carriers exhibit immunomodulatory effects of their own, either through their surface structure or their interaction with intestinal mucosa and microbiota. For instance, chitosan-based nanoparticles have been shown to stimulate mucosal immunity and improve gut barrier integrity while simultaneously enhancing the delivery of encapsulated omega-3 fatty acids or polyphenols [45,46]. This dual-function carrier and bioactive illustrates the potential of biomimetic encapsulation platforms as multifunctional nutraceutical interventions. In addition to oral delivery, biomimetic systems are being explored for alternative administration routes such as transdermal, nasal, and sublingual delivery, further expanding their application scope. Additionally, nanoemulsion-based systems that mimic skin lipids have shown promise in delivering fat-soluble vitamins and terpenoids through dermal layers, enhancing skin penetration and bioavailability without the use of chemical enhancers [47]. From a clinical translation standpoint, the increased bioefficacy of encapsulated nutraceuticals may reduce required dosing, enhance patient compliance, and lower cost-per-effect ratios, important considerations in both preventive nutrition and adjunctive therapeutic contexts. As personalized and functional nutrition continue to evolve, biomimetic encapsulation strategies are poised to play a central role in optimizing the theranostic potential of nutraceuticals.

## 4. Functional Principles and Mechanisms of Biomimetic Systems

The development of advanced nutraceutical delivery platforms is increasingly driven by biomimetic principles that take inspiration from natural processes and biological materials. Among these, bio-inspired and biogenic synthesis techniques have emerged as key strategies for generating nanocarriers that are both functional and environmentally compatible. These approaches draw upon plant metabolites, microbial enzymes, and natural polymers to produce nanoparticles capable of enhancing the stability, absorption, and therapeutic performance of bioactive compounds. The field of bionanomedicine has witnessed significant advances with the rise in bio-inspired synthesis techniques and biogenic nanomaterials, which emulate nature’s precise and sustainable methods for constructing functional nano-scale structures. Unlike conventional chemical or physical methods that often involve toxic solvents, high energy consumption, or expensive reagents, bio-inspired synthesis relies on natural reducing agents, biomolecular templates, and ecologically benign pathways to fabricate nanostructures with enhanced biocompatibility and functionality [48]. These attributes are particularly advantageous in the development of nutraceutical delivery systems, where safety, bioavailability, and biodegradability are of paramount importance.

Biogenic synthesis refers to the bottom-up fabrication of nanomaterials using biological entities such as plant extracts, microorganisms, enzymes, and biomolecules. These biological systems serve as both reducing and capping agents, enabling the controlled formation of nanomaterials with diverse morphologies, sizes, and surface properties. The process draws inspiration from nature’s own nanofactories, e.g., diatoms forming silica shells, or magnetotactic bacteria synthesizing magnetite nanoparticles demonstrating precise spatial control without the need for extreme conditions [49]. The underlying mechanisms often involve phenolics, flavonoids, proteins, terpenoids, and alkaloids acting as electron donors to reduce metal ions and stabilize the resulting nanostructures. For example, zinc oxide (ZnO) nanoparticles synthesized using Aloe vera extract have demonstrated improved antioxidant and antimicrobial properties, making them suitable for encapsulating polyphenolic nutraceuticals [50].

Moreover, green-synthesized metal and metal oxide nanoparticles such as silver (Ag), gold (Au), titanium dioxide (TiO_2_), and zinc oxide (ZnO) have garnered significant attention for their unique biofunctional properties, including antioxidant, antimicrobial, and enzyme-modulating activity. These nanoparticles are often synthesized using aqueous extracts of medicinal plants like Camellia sinensis (green tea), Azadirachta indica (neem), or Curcuma longa (turmeric), which act as both reducing and capping agents. Phytochemicals such as polyphenols, terpenoids, and alkaloids adsorbed onto the nanoparticle surface enhance biocompatibility and therapeutic synergy, allowing the nanocarrier itself to contribute bioactivity in addition to the encapsulated nutraceutical [51]. Their large surface-to-volume ratio, surface charge tunability, and redox activity make these nanoparticles ideal for modulating oxidative stress, stabilizing reactive nutraceuticals, and interacting with cellular membranes. For example, silver nanoparticles (AgNPs) synthesized via Azadirachta indica extract have been co-loaded with gallic acid, resulting in a nanocomposite with superior antibacterial, antioxidant, and radical-scavenging capacity compared to either component alone [52]. Such combinations are particularly promising for functional food and nutraceutical applications targeting inflammation, microbial overgrowth, or gut dysbiosis.

In parallel, biogenic carriers derived from natural polymers such as cellulose, starch, lignin, and chitosan can be structured into nanocapsules or nanogels using microbial fermentation, ionic gelation, or enzyme-assisted polymerization. These biopolymer-based systems are excellent for hydrophilic and water-sensitive compounds, including vitamin C, B-complex vitamins, and phenolic acids, due to their pH sensitivity, mucoadhesiveness, and controlled release behavior [53]. Their biodegradability and compatibility with the gastrointestinal environment make them attractive for oral nutraceutical formulations, especially when protection from gastric degradation and release in the intestinal tract is required. Hybrid nanocarriers, combining the functionality of plant-derived inorganic nanoparticles with biopolymer or lipid coatings, offer additional performance benefits through structural reinforcement, enhanced encapsulation efficiency, and synergistic bioactivity. For instance, copper oxide (CuO) nanoparticles synthesized using Ocimum sanctum (holy basil) extract and subsequently coated with casein or lecithin have demonstrated improved stability and sustained release of lipophilic compounds such as omega-3 fatty acids and curcumin. These systems capitalize on the bioactivity of the core nanoparticle, the stabilizing capacity of the protein/lipid shell, and the controlled-release behavior of the encapsulated compound, creating multifunctional platforms for therapeutic nutrition [54].

In addition to plant-mediated synthesis, several microorganisms, including fungi (*Aspergillus* and *Trichoderma*), bacteria (*Bacillus subtilis* and *Pseudomonas*), and yeasts (*Saccharomyces cerevisiae*), have been employed in the in situ biosynthesis of nanoparticles. These routes are highly effective for producing selenium, magnesium, and iron-based nanocarriers, which have been shown to improve the bioavailability, stability, and targeted delivery of trace elements and co-loaded bioactives. For example, selenium nanoparticles synthesized by probiotic strains such as Lactobacillus not only enhance antioxidant defenses but also support gut microbiota modulation, making them suitable for synbiotic applications [55]. Moreover, enzyme-assisted biosynthesis using enzymes such as nitrate reductases, peroxidases, and laccases has emerged as a scalable, precise, and environmentally friendly approach to nanoparticle fabrication. These biocatalytic platforms provide excellent control over particle size distribution, crystallinity, and morphology, all of which are critical for ensuring efficient cellular uptake, tissue penetration, and nutrient transport. Unlike traditional chemical reduction methods, enzyme-based synthesis enables the fine-tuned engineering of surface characteristics under mild, aqueous conditions, supporting greener manufacturing and compatibility with sensitive nutraceutical compounds [56].

Further on, biogenic nanomaterials offer a combination of biological functionality, eco-compatibility, and therapeutic enhancement, making them especially well-suited for nutraceutical delivery applications. A defining feature of these materials is their inherent bioactivity, derived from the phytochemicals or microbial metabolites used during synthesis. This can result in synergistic effects, where both the nanocarrier and the encapsulated nutraceutical contribute to therapeutic outcomes. For instance, metal oxide nanoparticles such as ZnO and SeNPs synthesized via plant or microbial routes not only serve as delivery vehicles but also exhibit antioxidant, anti-inflammatory, and antimicrobial properties, traits that amplify the effectiveness of encapsulated compounds such as curcumin, quercetin, or omega-3 fatty acids [57]. These materials have shown promising results in enhancing cellular uptake, reducing oxidative stress markers, and improving intestinal barrier function in preclinical models. Another functional advantage lies in the surface chemistry of biogenic nanoparticles, which often carries biofunctional groups (e.g., hydroxyl, carboxyl, and amine) from the capping biomolecules. These groups can facilitate mucoadhesion, receptor targeting, and endocytosis, enabling more effective interaction with gastrointestinal or epithelial tissues [58]. In some cases, the natural ligands present on the nanoparticle surface allow for passive or active targeting of inflamed or diseased tissues, such as inflammatory bowel disease or metabolic dysfunction.

Furthermore, the controlled and sustained release behavior of biogenic carriers improves the pharmacokinetics of many nutraceuticals. The natural polymer matrices such as lignin, chitosan, or starch used in these systems often degrade gradually in response to pH or enzymatic activity, allowing for site-specific and prolonged delivery in the gastrointestinal tract. Importantly, many of these nanomaterials are generally recognized as safe and biodegradable, minimizing the risk of cytotoxicity or environmental accumulation, which is a concern with some chemically synthesized nanoparticles [59]. As the field advances, integrating multifunctionality such as antioxidant action, microbiota modulation, and immune support into a single biogenic nanocarrier is emerging as a key strategy for next-generation nutraceutical therapies.

**Figure 2:**
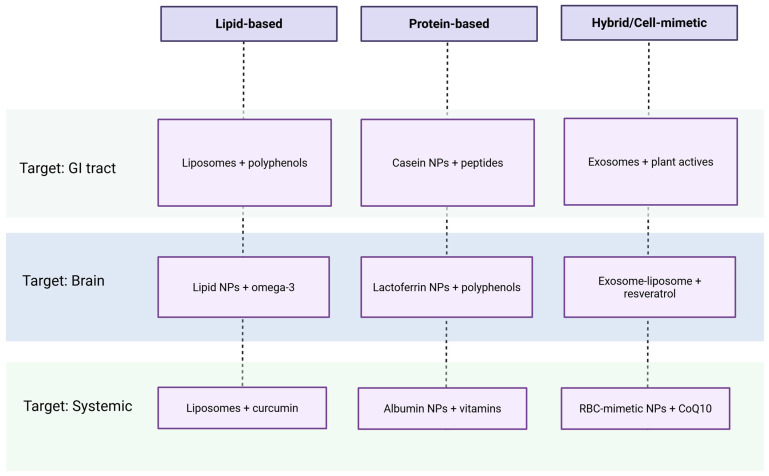
Classification of biomimetic delivery systems by material type, biological target, and bioactive compound.

Despite their promise, bio-inspired and biogenic systems face several translational hurdles. Standardization of synthesis protocols, batch-to-batch reproducibility, long-term stability, and scalability for industrial production remain major challenges. Furthermore, regulatory ambiguity surrounds their classification as food-grade or pharmaceutical-grade materials, complicating their path to market approval [60]. Toxicological evaluations are also essential, particularly when dealing with metal-based nanoparticles, to assess their safety in chronic dietary applications. The integration of multi-omics tools, including metabolomics and transcriptomics, is expected to improve our understanding of the biological behavior of these materials in complex physiological systems. Given the wide range of biogenic and bio-inspired nanomaterials discussed—spanning inorganic nanoparticles, biopolymer matrices, and hybrid structures—there is a growing need to categorize these systems not only by their composition but also by their functional intent. The diversity of encapsulated compounds, routes of administration, and target sites makes it essential to frame these strategies within a practical design logic. To address this, we introduce a classification matrix that maps representative biomimetic delivery systems according to three key dimensions: material type, biological target, and class of bioactive compound. This framework helps distill complex design decisions into an accessible visual overview (Figure 2).

## 5. Biomimetic Strategies for Enhanced Bioavailability

### 5.1. Challenges in Nutraceutical Bioavailability

Many nutraceutical compounds exhibit suboptimal bioavailability owing to issues such as poor water solubility, instability in the gastrointestinal (GI) tract, and rapid metabolic elimination [61,62]. For instance, curcumin and resveratrol—prototypical dietary polyphenols—suffer from low oral absorption and extensive first-pass metabolism, resulting in only trace levels reaching systemic circulation or target tissues [63,64]. These limitations have spurred the development of biomimetic delivery strategies in bionanomedicine aimed at preserving and enhancing nutraceutical bioavailability. Biomimetic nanocarriers leverage designs inspired by natural biological systems—such as cell membranes, lipoproteins, or extracellular vesicles—to protect sensitive nutraceuticals from degradation, improve their transport across biological barriers, and promote efficient uptake into cells. By emulating the body’s own delivery mechanisms, these advanced systems can significantly improve the pharmacokinetic profile and efficacy of nutraceuticals, as shown in numerous recent studies.

### 5.2. Lipid-Based Nanocarriers: Mimicking Natural Fat Transport

One major approach involves lipid-based nanocarriers like liposomes, nanoemulsions, micelles, and lipid nanoparticles, which mimic the composition and structure of biological membranes or dietary fat droplets. Such carriers create a biocompatible, often food-grade, environment that solubilizes hydrophobic nutraceuticals and shields them from harsh conditions. For example, oil-in-water nanoemulsions can dissolve large amounts of lipophilic compounds and protect them from hydrolysis and enzymatic degradation under GI conditions [65]. Encapsulation in these lipidic nanostructures prevents premature breakdown in the stomach or by intestinal enzymes, allowing more of the active compound to remain intact for absorption [66]. An emulsified or micellar form of a nutraceutical often shows superior uptake compared to the bulk crystalline form; encapsulating omega-3 fatty acids in nanoemulsion droplets, for instance, markedly increases their dispersion and surface area for digestion, leading to faster and higher absorption than unencapsulated oils [67]. Likewise, curcumin formulated in liposomes or polymeric micelles demonstrates greatly improved bioavailability. In one comparative study, a phospholipid complex of curcumin (a “phytosome” that mimics natural phospholipid carriers) achieved a ~3.4-fold higher absorption than plain curcumin, while a curcumin micellar formulation yielded a remarkable 9-fold increase in bioavailability [68]. These lipid-based nanocarriers often facilitate nutraceutical uptake via the lymphatic pathway as well, bypassing first-pass metabolism in the liver. By recreating the transport mechanisms of dietary lipids (e.g., chylomicron formation and membrane fusion), liposomal and lipid nanoparticle systems can not only enhance the fraction absorbed but also prolong the circulation time of nutraceuticals in vivo [69].

### 5.3. Cell Membrane-Coated Nanoparticles for Targeted Delivery

The net result is higher plasma exposure and greater tissue distribution of the bioactives, translating into enhanced biological effects. Indeed, a recent review of clinical trials with nano-formulated curcumin reported increases in relative bioavailability ranging from 9-fold up to 185-fold over unformulated curcumin, underscoring how profoundly nanotechnology can improve nutraceutical pharmacokinetics [70].

Another cutting-edge strategy is the use of cell membrane-coated nanoparticles (CMNPs)—a biomimetic platform that cloaks synthetic nanoparticle cores with natural cell membranes. This approach endows nanoparticles with a biological “cloak” derived from cells such as red blood cells (RBCs), leukocytes, platelets, or even cancer cells [71]. Coating nanoparticles with an RBC membrane, for example, presents them to the immune system as if they were native erythrocytes, possessing self-markers (e.g., CD47) that inhibit phagocytosis. Consequently, these biomimetic carriers can evade immune surveillance and remain in circulation far longer than uncoated particles [72]. Prolonged circulation increases the likelihood of the nanocarriers reaching and accumulating in target tissues before being cleared. In addition, the source of the membrane can impart targeting capabilities: nanoparticles wrapped in membranes from immune cells (like macrophages or T-cells) tend to home to inflamed or tumorous tissue by mimicking the natural trafficking of leukocytes to sites of injury or disease [73]. Similarly, cancer cell membrane-coated nanoparticles exhibit homotypic binding, a phenomenon where cancer-derived membranes can fuse with or adhere to membranes of the same cancer type, thereby guiding the payload back to the tumor microenvironment [74]. One study demonstrated that camouflaging polymeric nanoparticles with cancer cell membranes improved their accumulation in tumor tissue and penetration into the tumor mass compared to conventional nanoparticles [75]. Overall, cell membrane coating is a powerful biomimetic tactic to elude early clearance and achieve site-specific delivery.

This biomimetic membrane-coating approach has been successfully applied to nutraceutical compounds to boost their bioavailability and therapeutic index. Erythrocyte-coated nanoparticles encapsulating curcumin (a notoriously poorly bioavailable nutraceutical) showed significantly enhanced performance in preclinical models. Guo et al. prepared curcumin-loaded PLGA nanoparticles cloaked in RBC membranes and observed that the coated nanoparticles evaded uptake by macrophages while exhibiting greater cellular uptake by target cancer cells than their uncoated counterparts [76]. By avoiding phagocytic clearance, the RBC-mimetic nanocarriers delivered more curcumin to the tumor site, resulting in potent anti-tumor activity in a murine cancer model with no observable systemic toxicity [76]. This illustrates how biomimetic carriers can protect nutraceutical payloads in the bloodstream and ferry them to the intended site of action.

Likewise, “leukosome” nanoparticles—coated with leukocyte membranes—have shown an ability to target inflamed endothelium and tumors by exploiting natural cell-adhesion pathways, thereby concentrating anti-inflammatory or anticancer nutraceuticals at disease sites [77]. By mimicking platelets, another study used platelet membrane-cloaked nanocarriers to deliver antioxidants to damaged vasculature, taking advantage of platelets’ intrinsic affinity for vascular injury sites [78], although applications in nutraceutical contexts are still emerging. These examples underscore that cell membrane camouflage not only prolongs systemic retention but also directs nanocarriers to specific tissues, addressing both the bioavailability and targeting challenges.

### 5.4. Exosomes and Extracellular Vesicles as Nature’s Nanocarriers

Exosomes and other extracellular vesicles (EVs) represent another biomimetic delivery avenue that has gained traction for nutraceuticals. Exosomes are nano-scale vesicles naturally secreted by cells to transport biomolecules for intercellular communication. They possess a lipid bilayer enriched with membrane proteins and adhesion molecules, and can inherently cross biological barriers such as the intestinal epithelium, blood–brain barrier, and placenta [79]. Because exosomes are endogenously derived, they are highly biocompatible and less immunogenic, making them attractive as “nature’s nanoparticles” for delivering bioactives.

Researchers have started isolating exosomes from sources like milk or plants and loading them with nutraceutical compounds to exploit their stability and targeting properties. Notably, milk-derived exosomes have been shown to protect polyphenols from metabolism and efflux, dramatically improving their bioavailability and bioactivity [80]. Previous authors demonstrated that curcumin and resveratrol encapsulated in bovine milk exosomes were shielded from rapid degradation and achieved higher tissue levels than the free compounds. In that study, exosome-encapsulated curcumin and resveratrol, delivered intravenously, rapidly accumulated in breast tissue at nanomolar concentrations sufficient to exert potent antiproliferative effects on breast cancer cells, whereas equivalent doses of free curcumin or resveratrol did not reach detectable levels in the tissue [80]. The exosomal formulation markedly enhanced the nutraceuticals’ cytotoxic impact on cancer cells while sparing normal cells, suggesting a combination of improved delivery and intrinsic targeting.

Mechanistically, the exosomes were found to enter cancer cells mainly via clathrin-mediated endocytosis and avoid expulsion by ATP-dependent efflux transporters (ABC pumps) [80]. By acting as Trojan horses, milk exosomes smuggled the nutraceuticals into cells and bypassed the resistance mechanisms that often limit intracellular drug accumulation [80]. These findings exemplify how harnessing natural vesicles can overcome multiple barriers: exosomal carriers can traverse endothelial and cellular barriers, evade metabolic enzymes, and release their cargo directly inside target cells. Plant-derived exosome-like nanoparticles (for example, from ginger or grapes) are also under exploration for the oral delivery of nutraceuticals, since they may possess innate tropisms for certain gut or immune cells and are stable in the GI tract. Overall, leveraging exosomes and EVs merges nutrition with nanotechnology, potentially enabling the oral or systemic delivery of nutraceuticals in a form that the body “recognizes” and readily assimilates.

Despite their promising potential, the use of extracellular vesicles—particularly milk-derived and plant-derived exosome-like vesicles—faces several manufacturing challenges. These include low isolation yields, lack of standardized large-scale purification protocols, and variability between production batches. Additionally, ensuring vesicle purity, stability during storage, and consistent bioactivity remains a bottleneck for clinical and commercial applications. Overcoming these limitations is essential for the successful translation of EV-based delivery systems into regulated nutraceutical products [81].

### 5.5. Hybrid and Bio-Inspired Nanomaterials

In tandem with these sophisticated delivery vehicles, researchers are also designing hybrid and bio-inspired nanomaterials to improve nutraceutical bioavailability. *Hybrid nanocarriers* combine components (inorganic, polymeric, and lipidic) to achieve synergistic functionalities, often inspired by natural structures. For example, lipid–polymer hybrid nanoparticles have been developed where a polymer core provides structural integrity and controlled release, while a lipid shell offers a biomimetic interface for better biocompatibility and interaction with biological membranes.

Such hybrids can simulate lipoprotein particles or viral capsids, enhancing stability in circulation and cellular uptake. In one study, curcumin was loaded into a lipid–polymer hybrid nanoparticle functionalized with targeting ligands, which resulted in improved oral absorption and brain delivery of curcumin in an Alzheimer’s disease model (by mimicking the way certain natural nanoparticles cross the blood–brain barrier) [82]. Another innovation is biopolymer-based nanocarriers using naturally derived materials like chitosan, alginate, or dextran to encapsulate nutraceuticals. These materials are biodegradable and often mucoadhesive, meaning they can stick to the mucosal lining of the gut and prolong the residence time of the nutraceutical, thereby facilitating greater uptake. Chitosan in particular, a cationic polysaccharide, has been widely used to coat or form nanoparticles for oral nutraceutical delivery [83]. Chitosan’s positive charge enables it to adhere to negatively charged mucosal surfaces and transiently open tight junctions between intestinal cells, increasing paracellular transport of molecules [84]. It also protects encapsulated compounds as they transit through the acidic stomach environment. Encapsulating polyphenols like epigallocatechin gallate (EGCG) or anthocyanins in chitosan or pectin-based nanoparticles has been shown to improve their stability during digestion and modulate their release for absorption in the intestine [85]. For instance, EGCG (a green tea catechin) loaded in chitosan nanoparticles resisted degradation in the GI tract and maintained a higher fraction of bioactive form that could be absorbed, while also exhibiting increased anti-tumor activity in cell culture compared to free EGCG [86]. These bio-based carriers thus act as protective vehicles, ensuring that a meaningful dose of the nutraceutical survives long enough to be absorbed and exert biological effects.

### 5.6. Advancements in Pharmacokinetics and Therapeutic Impact

Crucially, biomimetic delivery systems do not merely improve absorption in the gut, but can also enhance the distribution and cellular uptake of nutraceuticals throughout the body. Traditional nutraceutical supplements often have “erratic bioavailability” and fail to achieve effective concentrations in target organs [87]. In contrast, nanoparticle-mediated delivery can be engineered for site-specific release or accumulation. Targeted delivery may involve functionalizing nanocarriers with ligands (such as antibodies, peptides, or vitamins) that recognize receptors on specific cell types, thereby mimicking the lock-and-key targeting seen in biological processes [88]. As an example, folic acid (a vitamin) is sometimes attached to liposomes or polymeric nanoparticles to exploit the overexpression of folate receptors on cancer cells, enhancing the uptake of encapsulated nutraceuticals in tumors. Similarly, the incorporation of cell-penetrating peptides—short peptides derived from viruses or proteins that naturally traverse cell membranes—can greatly improve the internalization of nutraceutical-loaded nanoparticles into cells [89]. In one report, grafting a TAT peptide (from HIV-1) onto curcumin-loaded liposomes increased their penetration into cells and deeper into tumor spheroids, resulting in higher intracellular curcumin levels and improved anticancer efficacy [90]. Through such bio-inspired modifications, nanocarriers can overcome cellular barriers that free nutraceutical molecules cannot easily cross.

Thanks to these advances in bionanotechnology, the pharmacokinetic profiles of nutraceuticals can be dramatically improved. Biomimetic carriers often confer a sustained release of the cargo, extending the nutraceutical’s presence in the bloodstream or at the site of absorption [72]. For example, porous polymer nanoparticles coated with RBC membrane achieved a more controlled 48 h release of curcumin compared to uncoated formulations [76]. Prolonging release and circulation time helps maintain therapeutic levels of the nutraceutical over longer durations. Furthermore, by preventing rapid metabolism, these strategies increase the effective half-life of compounds that would otherwise be quickly conjugated or excreted. The encapsulation of resveratrol in various nanocarriers (lipid nanoparticles, polymeric NPs, etc.) has been shown to lengthen its half-life and improve tissue distribution, addressing the issue that free resveratrol is metabolized within minutes in vivo [64,91].

Improved bioavailability directly correlates with enhanced bioefficacy in many cases. Higher plasma and tissue concentrations mean the nutraceutical can better exert its antioxidant, anti-inflammatory, or other health-promoting activities. In vivo studies have repeatedly demonstrated superior therapeutic outcomes with biomimetic nutraceutical formulations—such as stronger antioxidant effects, greater anti-inflammatory or anticancer responses, and beneficial modulation of biomarkers—compared to the native nutraceutical at the same dose [92,93]. For instance, nanostructured lipid carriers delivering curcumin not only raised its absorption but also yielded more pronounced reductions in inflammatory cytokines and oxidative stress in an animal model than free curcumin, indicating more effective bioactivity [92]. Likewise, the exosome-mediated delivery of curcumin and resveratrol enhanced their ability to induce cancer cell apoptosis and to overcome multidrug resistance mechanisms, effects that were not observed with the free nutraceuticals [93].

These improvements highlight how bionanomedicine can unlock the full potential of nutraceuticals by surmounting the biological barriers that limit their efficacy in conventional forms. In summary, biomimetic delivery strategies have ushered in a new era for enhancing the bioavailability of nutraceuticals. By mimicking natural carriers and biological interactions, researchers have created nano-scale delivery systems that significantly improve the stability, absorption, systemic transport, and targeted delivery of vitamins, polyphenols, fatty acids, and other bioactives. Liposomes and micelles provide protective, solubilizing vehicles that increase intestinal uptake; cell membrane-coated nanoparticles bestow stealth and disease-targeting capabilities; and exosomes offer a naturally optimized vesicle for crossing barriers and delivering payloads into cells. Hybrid and bio-inspired nanomaterials further expand the toolkit, allowing the customization of carriers to suit different nutraceutical profiles and therapeutic needs. The convergence of nutraceutical science with nanotechnology—especially leveraging biomimicry—has led to measurable gains in pharmacokinetics, with multiple studies reporting order-of-magnitude increases in circulating levels and improved tissue targeting for previously poorly bioavailable compounds [93]. These advances translate into enhanced efficacy, enabling nutraceuticals to more consistently realize their health benefits in vivo. While most findings to date are preclinical, some nano-formulated nutraceuticals have begun to reach clinical evaluation, and early results are promising [93]. Continued refinement of biomimetic nano-delivery systems, with attention to safety, scalability, and regulatory aspects, is expected to pave the way for next-generation nutraceutical therapies. In the broader context of nutritional health, such bionanomedicine approaches hold the potential to maximize the preventive and therapeutic impacts of natural compounds, bridging the gap between potent bioactivity in the lab and tangible benefits in the human body. Through biomimetic strategies for enhanced bioavailability, nutraceuticals can be delivered more effectively to where they are needed most, heralding improved outcomes in health promotion and disease management (Table 2).

## 6. Biomimetic Antioxidant Systems

Biomimetic antioxidant systems have emerged as a cutting-edge solution to mitigate oxidative stress, a molecular imbalance characterized by the accumulation of ROS that damages lipids, proteins, and DNA, contributing to the development of chronic diseases. Inspired by natural antioxidant defense mechanisms, these systems seek to optimize the delivery of antioxidant compounds through advanced technologies that improve their stability, bioavailability, and specificity in biological environments [94].

The basis of these systems lies in the imitation and refinement of the organism’s natural processes. Endogenous antioxidant enzymes, including superoxide dismutase (SOD), catalase, and glutathione peroxidase, are pivotal in neutralizing free radicals [95]. Utilizing these properties as a model, biomimetic systems are designed to develop innovative therapeutic solutions.

### 6.1. Biomimetic Catalytic Materials—Nanozymes and Their Role as Antioxidants

The advent of biomimetic catalytic materials, particularly nanozymes, has ushered in a paradigm shift in the realm of antioxidant systems design. These nanostructures are designed to emulate the enzymatic activity of natural antioxidants (SOD, catalase, and glutathione peroxidase) [96]. Their capacity to neutralize ROS with a level of efficiency comparable to (or in some cases superior to) that of natural enzymes has led to the recognition of these materials as a potentially transformative tool in the field of oxidative stress mitigation [97,98]. Oxidative stress has been implicated in the development of various pathologies, including neurological disorders, diabetes, and a wide range of cardiovascular diseases [99].

Among the most extensively studied nanozymes are cerium oxide nanoparticles (CeO_2_), which are distinguished by their distinctive regenerative capacity attributable to the reversible redox states of cerium ions (Ce^3+^/Ce^4+^) [100,101]. This mechanism enables the continuous catalytic scavenging of superoxide radicals and hydrogen peroxide, thereby prolonging their antioxidant activity over time. In recent studies, cerium oxide nanoparticles have been demonstrated to markedly reduce oxidative stress levels in neuronal tissues, impeding the accumulation of β-amyloid and delaying the progression of neurodegenerative processes [102,103].

Similarly, manganese oxide nanoparticles (MnO_2_) have garnered significant attention for their capacity to emulate the catalytic activity of catalase. These particles facilitate the decomposition of hydrogen peroxide into water and oxygen, thereby mitigating the oxidative damage that occurs in chronic inflammatory settings [104]. Previous research have explored the use of MnO_2_ in therapeutic platforms integrated with biological sensors, allowing for the real-time detection of ROS and activation of catalytic activity depending on the needs of the microenvironment [105,106].

Another promising approach involves the functionalization of nanozymes with bioactive molecules to enhance their specificity and efficacy. The conjugation of iron oxide nanoparticles (Fe_3_O_4_) with polyphenols, such as tannic acid, enhances their antioxidant capacity while also improving their stability within biological environments, including the gastrointestinal tract [107,108]. These functionalized nanozymes are currently being investigated as therapeutic agents in inflammatory bowel diseases, where elevated levels of ROS contribute to the dysfunction of biological barriers.

From a biomimetic standpoint, these nanozymes emulate natural activities and optimize them by offering unique properties, including resistance to enzymatic degradation. In addition, they can be customized to address specific therapeutic needs [109]. In this line, the precise engineering of nanoparticle size and surface charge enables their selective accumulation in affected tissues, such as the brain or regions exhibiting elevated levels of oxidative stress [110,111]. Furthermore, their capacity to interface with other biomimetic systems, such as antioxidant-loaded liposomes, engenders prospects for combined therapeutic modalities with augmented efficacy [112].

Despite their potential, the clinical application of nanozymes faces significant challenges. Key areas requiring intensive research include long-term biocompatibility, potential immunological impact, and controlled removal from the body. Furthermore, the interaction of these nanozymes with complex biological systems, such as the gut microbiome, raises questions about their influence on microbiological balance. Nevertheless, advancements in bioengineering and nanotechnology are establishing the foundation to address these limitations, rendering nanozymes a promising instrument in personalized antioxidant medicine.

### 6.2. Encapsulation of Antioxidant Compounds—Improved Stability and Bioavailability

The encapsulation of antioxidant compounds is a critical strategy for overcoming the inherent limitations of these agents. These limitations include their low solubility in aqueous media, susceptibility to chemical or thermal degradation, and limited ability to cross biological barriers [113]. This biomimetic approach facilitates the design of advanced systems that protect antioxidants during transport and release in biological environments, thereby ensuring their therapeutic efficacy.

Lipid vesicles, including liposomes and solid lipid nanoparticles, have demonstrated remarkable efficacy as platforms for the delivery of antioxidants such as resveratrol, vitamin E, and curcumin. These biomimetic structures have been shown to mimic the natural characteristics of cell membranes, facilitating the integration of the antioxidant into specific tissues and increasing its bioavailability [114,115]. Recent studies have shown that encapsulating curcumin in liposomes improves its stability by up to five times compared to its free form, increasing its ability to counteract oxidative stress [116,117].

Another salient strategy involves the utilization of biodegradable polymers, including polylactic-co-glycolic acid (PLGA) derivatives and chitosan. These polymers have been shown to protect antioxidants from adverse conditions, such as the acidic pH of the gastrointestinal tract [118,119]. Their capacity to release compounds in a regulated manner ensures prolonged availability in specific tissues [119]. In this regard, the encapsulation of chitosan or vitamins in PLGA microparticles has demonstrated substantial enhancements in the stability of the compound and its capacity to combat free radicals in recent studies [120,121].

In recent years, halloysite nanotubes have emerged as innovative tools for encapsulating antioxidants due to their cylindrical structure and inherent biocompatibility. These nanotubes protect the encapsulated compounds against degradation and enable targeted release through chemical modifications on their surface [122]. Previous studies have demonstrated that the use of functionalized nanotubes to deliver ascorbic acid has led to a more than 250% increase in its antioxidant activity, underscoring its promise for medical applications [123,124].

The combination of encapsulation systems with stimuli-activated technologies has elevated the therapeutic potential of antioxidants. In this approach, the encapsulation of nanoparticles is such that their contents are released in response to specific changes in the microenvironment, such as pH, temperature, or ROS [125]. This targeted release enables the optimization of antioxidant efficacy while minimizing adverse effects on unaffected tissues. In this line, pH-sensitive nanoparticles engineered to release vitamin E in acidic environments have demonstrated significant potential in protecting against oxidative damage in inflammatory processes [126,127].

Beyond traditional technologies, the development of multilayer biomimetic systems, integrating protective and functional layers, signifies an emerging frontier in the field of antioxidant encapsulation. These structures have been shown to mimic the protective properties of cell membranes, incorporating endogenous antioxidants in an environment that favors their prolonged and targeted action. Specifically, glutathione-enriched multilayer membranes have demonstrated efficacy in preventing lipid peroxidation in cellular oxidative damage [128].

Antioxidant encapsulation is also being adapted for specific applications in the gut–brain axis. For instance, antioxidants encapsulated in lipid vesicles engineered to withstand the conditions of the gastrointestinal tract have demonstrated efficacy in neuronal protection through the modulation of neuroinflammation associated with oxidative stress [129,130]. These innovations hold promise in the management of neurodegenerative diseases, where oxidative damage plays a pivotal role.

### 6.3. Therapeutic Applications and Future Potential

Biomimetic antioxidant systems have demonstrated their ability to intervene in the molecular processes involved in a specific method, offering targeted and effective solutions in a variety of clinical scenarios. A particularly promising field of research is that of neurodegenerative diseases, where oxidative damage has been identified as a critical factor in neuronal death and cognitive decline. In this line, cerium oxide-based nanozymes have demonstrated a substantial impact on reducing oxidative stress in the brain, enhancing neuronal survival, and delaying the accumulation of β-amyloid and α-synuclein, proteins associated with these pathologies [131,132]. Furthermore, biomimetic membranes, engineered to fortify the blood–brain barrier, have demonstrated efficacy in impeding the infiltration of proinflammatory molecules into the CNS, thereby attenuating neuroinflammation and enhancing cognitive function [133].

Another area of clinical interest lies in cardiovascular diseases, where oxidative stress is a central mediator of vascular damage and endothelial dysfunction. Stimulus-triggered systems, engineered to release antioxidants in response to elevated ROS, have demonstrated efficacy in preventing lipid oxidation and safeguarding cell membranes within vascular barriers [134]. pH-sensitive nanoparticles have been utilized for the controlled release of vitamin C in ischemic environments, resulting in a substantial enhancement in the recovery of damaged tissues following cardiovascular events [135].

In addition, biomimetic antioxidant systems are being investigated as preventative measures for high-risk populations. Formulations designed for sustained release, such as liposomes enriched with vitamin E and glutathione, have demonstrated potential in preventing cellular damage in athletes subjected to extreme physical stress, as well as in individuals exposed to environments with high levels of pollution.

In the context of biomimetic antioxidant systems, understanding the technological transition from synthetic to biomimetic nanoparticles is essential. Although biomimetic carriers offer enhanced biocompatibility and targeted antioxidant delivery, their development presents significant formulation and manufacturing challenges. Table 3 summarizes various nanoparticle platforms used in nutraceutical delivery, highlighting the specific benefits they offer, the difficulties associated with implementing biomimetic strategies, and the current biomimetic solutions along with their limitations. This overview contextualizes the innovation landscape and clarifies the gaps that remain in optimizing antioxidant delivery through biomimetic nanotechnology.

## 7. Anti-Inflammatory Biomimetic Formulations

Inflammation is an essential physiological process that the body uses to defend against infection or injury. It is a complex process involving interactions between immune cells, chemical mediators, and affected tissues. While acute inflammation is vital for tissue recovery and repair, chronic inflammation poses a grave threat to health, contributing to the development and progression of various diseases, including autoimmune, cardiovascular, metabolic, and neurodegenerative diseases [136,137]. In this regard, anti-inflammatory biomimetic formulations have emerged as a sophisticated solution to address inflammatory imbalance in a targeted and efficient manner, emulating and optimizing the body’s natural mechanisms.

The design of these formulations is based on biomimetic principles that seek to replicate the biological processes involved in the resolution of inflammation and the restoration of homeostasis. Bioactive molecules, including polyphenols, omega-3 fatty acids, and peptides derived from natural proteins, have emerged as the primary agents employed in these formulations due to their acknowledged anti-inflammatory properties [138]. In this regard, polyphenols, found in compounds such as resveratrol and quercetin, have been shown to impede the activation of nuclear factor κB (NF-κB), a pivotal pathway in proinflammatory signaling [139]. Similarly, omega-3 fatty acids, such as eicosapentaenoic acid (EPA) and docosahexaenoic acid (DHA), have been shown to modulate the synthesis of prostaglandins and leukotrienes, thereby promoting resolving processes that limit inflammation [140,141].

However, the inherent limitations associated with the stability and bioavailability of these compounds within biological environments have spurred the development of advanced biomimetic systems for their administration. Functionalized nanoparticles exemplify a pivotal innovation in this domain, serving as a secure and effective carrier for the delivery of anti-inflammatory agents [142,143]. These structures are engineered to release their contents exclusively in response to specific stimuli from the inflamed microenvironment, such as acidic pH, elevated temperature, or elevated levels of ROS. In this line, ROS-responsive nanoparticles loaded with curcumin have demonstrated encouraging outcomes in preclinical models of rheumatoid arthritis, exhibiting the capacity to reduce localized inflammation and minimize adverse effects on healthy tissues [144,145].

In addition, smart hydrogels have demonstrated their efficacy as therapeutic platforms within this domain. These materials possess the capacity to dynamically adapt to the inflammatory environment, releasing anti-inflammatory agents in a sustained manner to maximize their efficacy. In clinical applications, hydrogels loaded with bioactive molecules, such as hyaluronic acid, have proven effective in reducing chronic inflammation in joint tissues affected by osteoarthritis, providing long-lasting relief and promoting tissue regeneration.

Another innovative approach involves the use of biomimetic membranes that emulate the protective properties of natural biological barriers, such as the extracellular matrix and cell membranes [146]. These structures serve as platforms for the delivery of anti-inflammatory compounds and the limitation of proinflammatory mediator progression in affected tissues [147]. Recent studies have demonstrated that membranes enriched with omega-3 fatty acids can effectively reduce vascular inflammation and enhance endothelial regeneration. This multifaceted strategy offers a comprehensive approach to addressing inflammation, leveraging the potential of biomimetic membranes to optimize the delivery and efficacy of therapeutic agents [148].

The therapeutic applications of anti-inflammatory biomimetic formulations extend to a wide variety of chronic pathologies. In autoimmune diseases, such as multiple sclerosis and inflammatory bowel disease, these formulations have been shown to modulate immune balance by promoting the expansion of regulatory T lymphocytes (Tregs) and suppressing proinflammatory cytokines such as IL-6 and TNF-α [149,150]. In the domain of metabolic diseases, nanoparticles that are functionalized to release bioactive compounds in inflamed adipose tissues have shown promising results in the management of insulin resistance and dyslipidemia, thereby improving the metabolic health of patients [151]. In the context of neurodegenerative diseases, these formulations have demonstrated their ability to cross the blood–brain barrier and reduce neuroinflammation by the targeted delivery of resolving agents in the CNS [152].

The future potential of these formulations extends to the domain of personalized medicine, where the integration of emerging technologies, such as artificial intelligence, allows for the design of treatments that are tailored to each patient’s unique characteristics. Specifically, machine learning-based algorithms have the capacity to assess individual immunological and metabolic profiles, thereby facilitating the optimization of anti-inflammatory agent selection and dosage. This, in turn, enhances therapeutic efficacy while concomitantly reducing adverse effects.

It is evident that biomimetic anti-inflammatory formulations signify a substantial advancement in the management of chronic inflammation and its repercussions for human health. By integrating natural ingredients with advanced delivery and controlled-release technologies, these formulations offer a safe, effective, and personalized approach to the treatment of inflammatory diseases, thereby marking a significant milestone in translational and preventive medicine.

## 8. Chronic Disease Management Through Biomimetic Nutraceuticals

Chronic diseases such as diabetes, cardiovascular disorders, cancer, and neurodegenerative conditions represent a growing global health challenge. In this context, biomimetic nutraceuticals have emerged as a novel and promising approach to support prevention and treatment strategies by mimicking biological processes and enhancing the bioavailability of active compounds. Biomimetic nanotechnology enables the design of more efficient nutraceutical delivery systems, optimizing absorption and controlled release [153]. For instance, Jimenez-Jimenez exposed how nanoparticles coated with biological membranes (e.g., red blood cells or platelets) can target specific tissues affected by chronic inflammation or tumor growth [154]. Another study showed that biomimetic liposomes and artificial extracellular vesicles may also be used to encapsulate antioxidants, polyphenols, and omega-3 fatty acids, boosting their anti-inflammatory and neuroprotective effects [155]. Thus, nutraceuticals—bioactive compounds derived from plants, foods, or microbial sources—offer significant health benefits with fewer side effects compared to traditional pharmaceuticals. While early food analysis focused primarily on taste and basic nutrition, it is now well established that components such as probiotics, antioxidants, and phytochemicals play a critical role in preventing chronic diseases, enhancing overall health, delaying aging, and increasing lifespan. These compounds are found in various forms, including dietary supplements, functional foods, medical foods, and pharmaceuticals.

Knowing this, recent studies have demonstrated that biomimetic delivery systems for nutraceuticals can effectively reduce inflammatory markers, improve lipid profiles, and support cellular regeneration—advancing the potential for personalized nutritional medicine in the management of chronic conditions [153].

### 8.1. Cardiovascular and Nervous System Disorders

Recent advances have focused on the application of nutraceuticals in the prevention and treatment of cardiovascular and nervous system disorders, where targeted delivery systems show great promise. In this regard, several recent studies have explored the potential of nutraceuticals in the treatment of neurological disorders. For instance, Makkar et al. reviewed how nutraceuticals can help prevent and manage neurodegenerative diseases by modulating molecular pathways involved in neurodegeneration [156]. Additionally, Liao et al. analyzed recent advances in biomimetic membrane materials for treating central nervous system disorders, highlighting their potential to enhance drug delivery and support neural regeneration [157]. Finally, Dadhania et al. investigated how nutraceuticals may counteract neurodegeneration by modulating mitochondrial dysfunction, intracellular calcium overload, oxidative stress, and inflammation [158]. Collectively, these studies underscore the growing interest in nutraceuticals as complementary strategies within personalized medicine for managing neurological diseases.

Regarding cardiovascular diseases, Antonella Antonelli et al. investigated innovative nutraceutical therapies for fibromyalgia, emphasizing the use of plant-derived flavonoids and novel delivery systems, such as red blood cell encapsulation, to enhance therapeutic efficacy and reduce drug-related side effects [159]. Similarly, Roberta Macrì and colleagues (2024) reviewed the role of lifestyle modifications, dietary interventions, and nutraceutical supplementation in the prevention and treatment of heart failure, highlighting their potential while calling for further clinical validation [160]. Additionally, Sindhu C. Pillai and her team investigated the use of nanomaterials for the delivery of nutraceuticals in the treatment of atherosclerosis. Their study highlights how nanoparticles can enhance the delivery and efficacy of bioactive compounds, offering a promising strategy to target therapies to specific atherosclerotic sites with high therapeutic efficiency and reduced side effects [5]. These findings reinforce the growing relevance of nutraceuticals as complementary tools within the framework of personalized medicine for chronic disease management.

### 8.2. Metabolic Disorders

Recent studies have also highlighted the potential of nanotechnology-enhanced nutraceuticals in managing metabolic disorders such as obesity and metabolic syndrome. For instance, Lamia A. Heikal and colleagues developed pterostilbene-loaded chitosan nanoparticles, which, when administered orally, demonstrated significant anti-obesity effects in high-fat diet-induced obese rats. The treatment led to sustainable weight loss with minimal side effects, attributed to improved bioavailability and efficacy of pterostilbene [161]. Similarly, Sahar Y. Al-Okbi et al. investigated nutraceuticals composed of chitosan, ferulic acid, and β-sitosterol, along with their nanoformulations, in a rat model of metabolic syndrome induced by a high fructose-high fat diet. The study found that these formulations improved lipid profiles, reduced insulin resistance, and alleviated liver steatosis. Notably, the nanoformulations exhibited superior efficacy compared to their conventional counterparts, suggesting that nanostructured delivery systems can enhance the therapeutic potential of nutraceuticals in metabolic syndrome management [162]. Recent research has highlighted the potential of biomimetic cellulose-based oral superabsorbent hydrogel (OSH) in preventing metabolic syndrome. Sylvestri et al. demonstrated that OSH treatment in mice functions as a protective dynamic exoskeleton within the gut, positively influencing intestinal tissue and altering gut microbiota composition. These changes contribute to mitigating factors associated with metabolic syndrome [163]. These findings underscore the promise of nanotechnology in enhancing the effectiveness of nutraceuticals for treating metabolic disorders, offering avenues for more efficient and targeted interventions with reduced adverse effects.

One of the key motivations for the shift toward biomimetic nanoparticle systems lies in their potential to reduce immunological risks associated with synthetic nanocarriers. PEGylated liposomes, such as Doxil, have been shown to induce hypersensitivity reactions due to pre-existing anti-PEG antibodies and are associated with the phenomenon of accelerated blood clearance (ABC) upon repeated administration. These effects can compromise therapeutic efficacy and increase systemic toxicity [164]. In contrast, biomimetic nanoparticles coated with natural membranes—such as erythrocytes, leukocytes, or exosomes—offer improved biocompatibility and are less likely to trigger complement activation or adaptive immune responses. By presenting ‘self’ markers to the host immune system, these carriers may reduce humoral risk and extend circulation time without inducing antibody-mediated clearance, particularly in chronic or repeated-use scenarios [164,165].

This safety-oriented advantage further strengthens the rationale for integrating biomimetic strategies into nutraceutical delivery systems, which represent a significant advancement in the prevention and management of chronic diseases. These technologies not only enhance the bioavailability and targeted delivery of bioactive compounds but also minimize side effects commonly associated with conventional pharmacological treatments. As the burden of chronic illnesses continues to rise globally, biomimetic nutraceuticals offer a promising avenue for safe, effective, and personalized interventions. Future research should focus on clinical validation, scalability of production, and regulatory frameworks to facilitate their translation from laboratory to clinical practice.

## 9. Risk-Based Strategies in Biomimetic Nanocarrier Development

### 9.1. Safety and Regulatory Considerations

Biomimetic nanocarriers for nutraceutical delivery offer innovative health benefits, but their adoption hinges on rigorous safety evaluation and clear regulatory oversight. Globally, regulators are working to adapt the existing frameworks to nano-scale materials, acknowledging that nanomaterials may behave differently than their bulk counterparts [166,167]. A coordinated international approach is emerging as agencies strive to harmonize definitions and risk assessment methods for nano-enabled foods. However, significant challenges remain in ensuring these novel delivery systems are safe, effectively regulated, and transparently communicated to consumers.

In parallel with regulatory oversight, design-based optimization has emerged as a valuable tool to guide the safe and effective development of biomimetic nanocarriers. This approach relies on computational modeling, artificial intelligence, and in silico simulations to refine nanoparticle attributes such as size, surface chemistry, release kinetics, and circulation time. By proactively adjusting these variables to match physiological targets or risk profiles, researchers can predict performance outcomes and mitigate potential safety concerns before clinical application. For instance, the circulation behavior and release performance of nanoparticles can be fine-tuned to reduce off-target exposure and enhance bioavailability in specific tissues. These strategies support a rational, risk-driven design logic that complements the existing regulatory frameworks and enhances the translational potential of complex nanocarriers [168].

### 9.2. Regulatory Frameworks in Major Regions

United States (FDA): In the U.S., nutraceuticals (marketed as dietary supplements or functional foods) are regulated under the existing food safety laws without a nano-specific statute. The FDA has not established a formal legal definition for “nanomaterial,” opting instead for a case-by-case evaluation of products involving nanotechnology [167]. Regulators apply general provisions of the Federal Food, Drug, and Cosmetic Act, meaning a nano-formulated ingredient is subject to the same requirements as its conventional form—with important caveats. The FDA’s 2014 guidance advises manufacturers that any “significant manufacturing process change—including nanotechnology” could affect a food ingredient’s identity or safety, potentially requiring new regulatory review [169]. In particular, the FDA warns that a substance generally recognized as safe (GRAS) in normal form may not automatically be GRAS at the nano-scale. For example, although bulk silver and titanium dioxide have had GRAS or authorized uses, their nanoparticulate versions demand renewed safety evidence. The FDA guidance explicitly states that safety evaluations for nano-ingredients cannot rely solely on data from larger-sized counterparts. Manufacturers are encouraged to consult the FDA early in development and may need to submit a new dietary ingredient notification or food additive petition if a nanoform ingredient is not already legally in use [169]. Overall, the FDA’s stance is that the existing food and supplement regulations are applicable to nanotechnology, but additional scientific data may be required to demonstrate safety at the nano-scale [167]. Post-market monitoring is also emphasized for any novel nano-based ingredient. This flexible, case-by-case approach reflects the FDA’s attempt to foster innovation while upholding safety, though some critics argue it leaves regulatory gaps. Indeed, consumer advocacy groups have noted that there is currently no specific U.S. regulation mandating how nanotech can be used in food, only non-binding guidelines [170]. This contrasts with more precautionary regimes elsewhere.

Europe (EFSA and EU Regulations): Europe has instituted more defined requirements for nano-enabled nutraceuticals, treating engineered nanomaterials in food as new substances that generally require pre-market authorization. The European Commission’s Novel Food Regulation (EU) 2015/2283 includes any “engineered nanomaterial” as a novel food ingredient, meaning nutraceutical products using nanocarriers likely fall under this rule and must undergo a safety assessment before commercialization [171]. Similarly, if nanotechnology is used in food additives or food contact materials, EU law demands evaluation by the European Food Safety Authority (EFSA) and regulatory approval prior to marketing. EFSA has developed detailed guidance for risk assessment of nanomaterials in the food and feed chain (updated in 2018 and 2021), which outlines how to characterize nanoparticle properties, perform toxicological studies, and assess exposure for nano-sized ingredients [172]. These guidelines recognize that nanomaterials may exhibit “small particle” behavior not seen in larger forms—for example, increased reactivity or novel bioavailability—and thus require specialized testing [173]. A concrete example of the EU’s precautionary approach is the case of titanium dioxide (E171). Formerly used as a pigment in foods and supplements, E171 often contains a fraction of nano-sized particles. In 2021, after reviewing new evidence on its particulate absorption and potential DNA damage, EFSA concluded that titanium dioxide “can no longer be considered safe as a food additive” [174]. The panel could not exclude genotoxicity and noted that although oral absorption of TiO_2_ particles is low, they can accumulate in the body over time. This opinion—driven largely by nanotoxicological concerns—led the European Commission to ban titanium dioxide in foods. The TiO_2_ decision highlights how European regulators respond to emerging safety data on nanomaterials, taking a cautionary stance when long-term risks (like particle buildup or organ penetration) cannot be ruled out. Europe has also been a leader in labeling and transparency for nanotech in food: since Regulation (EU) 1169/2011, any engineered nanomaterial in an ingredient list must be indicated by the word “nano” in parentheses [175]. This labeling requirement aims to inform consumers of the presence of nano-scale ingredients. No other major jurisdiction has yet mandated such nano-specific labeling on foods, making the EU’s approach distinctive in prioritizing consumer right-to-know. Overall, the EU framework—combining pre-market safety reviews, dedicated scientific guidance, and labeling rules—reflects a robust, precautionary strategy to govern nutraceutical nanocarriers.

Asia and Other Regions: Regulatory oversight in other parts of the world is evolving in parallel, often drawing from US and EU paradigms. In India, for instance, the Food Safety and Standards Authority of India (FSSAI) has issued guidance on the use of nanotechnology in food and agri-products under the existing Food Safety and Standards Act [171]. Indian regulations on food additives (FSSAI 2011) explicitly include those “derived from nanotechnology,” meaning nano-ingredients are subject to the same approval conditions as conventional additives. These steps ensure that nano-scale additives in foods or supplements in India undergo safety evaluation and comply with quality standards. China similarly requires that new food ingredients or additives, including nanoforms, pass safety assessments by authorities before entering the market. While China does not yet have a separate nanotech food law, its regulators have been active in research on nano-risk assessment and are revising food safety standards to address nano-scale contaminants and additives [167]. Japan and South Korea regulate nutraceuticals (e.g., health foods and functional foods) through the existing food safety and food additive frameworks; these countries have supported research on nanomaterial safety but generally handle nano-ingredients within the conventional approval processes (e.g., Japan’s Food Sanitation Act for additives and Korea’s functional food regulations). Notably, Japan’s regulatory agencies have been monitoring nanotechnology developments, and any novel nano-encapsulated ingredient likely would be reviewed as a new food additive or under the “Foods for Specified Health Uses (FOSHU)” system for safety. In Canada, authorities have taken steps similar to the EU: Health Canada requires that companies inform regulators if nanotechnology is used in a new food ingredient or packaging, and a scientific assessment is conducted case-by-case, albeit without a formal nano-specific law. Several Latin American countries are also observing international norms; for example, Brazil and Argentina currently evaluate nano-nutraceuticals under general food additive or supplement regulations, while participating in Codex Alimentarius discussions on nanotech in food. In summary, across Asia and South America, there is growing recognition that existing food safety frameworks must account for nanomaterials, either through explicit guidelines (as in India) or by interpreting general safety requirements to include nano-scale considerations. Regulators worldwide acknowledge the need to “keep pace” with rapid nanotechnology developments in the food sector [167], and international bodies like the OECD and FAO/WHO are facilitating the exchange of best practices. This global convergence is gradually leading to more uniform safety standards for biomimetic nanocarriers, even if the pace and stringency of regulations differ by country.

### 9.3. GRAS Status, Food-Grade Materials, and Biocompatibility

From a safety perspective, one advantage of many biomimetic delivery systems is that they are composed of food-grade, biocompatible materials. Lipid nanoparticles, protein-based carriers, and polysaccharide nanogels used for nutraceuticals often utilize substances that are traditionally “Generally Recognized As Safe” (GRAS)—for example, lecithin phospholipids, milk casein, chitosan, or alginate [173,176]. The use of inherently edible materials can facilitate regulatory acceptance. However, GRAS status at the bulk scale does not automatically guarantee safety at the nano-scale, as regulators and experts caution [172].

Nanosizing an ingredient can alter its absorption and interaction with the body. Thus, even a nano-encapsulated form of a common vitamin or herbal extract may require fresh toxicological scrutiny. Ensuring biocompatibility involves demonstrating that the nanocarrier itself (apart from the nutraceutical payload) is non-toxic, non-immunogenic, and degrades into safe byproducts. Researchers typically select natural or biomimetic components to minimize adverse reactions—for instance, using cell membrane-coated nanoparticles or exosome-mimicking vesicles that emulate biological surfaces [167]. Such strategies can improve biocompatibility and reduce immune recognition. Nonetheless, rigorous testing is needed to confirm that these carriers do not trigger unexpected effects. For example, if a nanocarrier is coated with a plant or animal cell membrane, regulators will ask whether any residual proteins or lipids could provoke allergies or immune responses [177].

The GRAS concept is being re-interpreted under nanotechnology: as the FDA has stated, manufacturers cannot assume a “generally recognized as safe” ingredient remains safe when engineered to the nano-scale without evidence [169]. In practice, companies developing nanoformulations must either rely on ingredients with long histories of safe use and show the nanoform is equivalent or conduct additional studies to affirm safety. Many nutraceutical nanocarriers thus use well-known food additives (e.g., corn starch, soy lecithin, or gelatin) in their construction to leverage existing safety data [166]. This safe-by-design approach—formulating with GRAS or food-approved materials—is a key strategy to meet regulatory expectations. Still, as nanocarriers often have novel properties (high surface area or novel structures), regulators remain cautious. To attain GRAS status or its equivalent, it is increasingly expected that companies provide nano-specific safety data. In sum, while biomimetic nanocarriers start with a biocompatible foundation, their safety must be proven and not merely presumed from traditional use.

### 9.4. Nanotoxicological Concerns and Long-Term Safety

Nanotoxicology has emerged as a critical aspect of evaluating nutraceutical delivery systems. Due to their small size, nanoparticles can exhibit unique biodistribution and biological interactions that raise important safety questions. A primary concern is whether ingested nanocarriers remain confined to the gastrointestinal tract (like conventional food particles) or translocate into tissues and organs [178]. Studies have shown that certain nanoparticles can cross gut epithelium or other barriers and accumulate in secondary organs like the liver, spleen, or brain [179].

For nutraceuticals, the extent of absorption of the carrier itself (not just the nutrient) needs careful assessment. Particle size and shape are known to influence toxicity: smaller or rod-like particles have a greater likelihood of cellular uptake and tissue penetration than larger or spherical ones [176]. If a nanocarrier is too small, it might enter the bloodstream and distribute systemically, which is not typical for food ingredients. This could potentially lead to unintended effects in distant organs or prolonged residence in the body. Indeed, chronic exposure studies (such as those on titanium dioxide or silicate nanoparticles) suggest that even low absorption can, over a long period, result in bioaccumulation and possible chronic toxicity [174].

Additionally, the surface properties of nanoparticles strongly affect their interaction with cells. Positively charged (cationic) nanoparticles, for instance, tend to have higher cellular uptake but also higher cytotoxicity due to membrane disruption, whereas neutral or negatively charged particles are often less acutely toxic [173]. This means that even if made of safe materials, a nanocarrier with a certain surface charge or coating could cause local irritation or cell stress. Researchers have correlated highly cationic nanoemulsions or dendrimers with increased cell damage and inflammation, underscoring the need to optimize surface chemistry for safety (Shatkin & Kim, 2015) [177].

Other nanotoxicological issues include oxidative stress and DNA damage: nanoparticles can catalyze reactive oxygen species or interfere with cellular components, leading to genotoxicity. EFSA’s concern over titanium dioxide’s genotoxic potential exemplifies this point [174]. Importantly, many biomimetic carriers (like liposomes or protein nanoparticles) have shown low inherent cytotoxicity in cell studies, but each new formulation must be evaluated to ensure it does not induce oxidative stress or inflammatory pathways in gut tissues [167].

Immunogenicity is another safety dimension. Biomimetic nanocarriers are often designed to evade the immune system (for example, using “self” markers or natural membranes), yet paradoxically, they could trigger immune reactions if recognized as foreign. Some nanocarriers incorporate peptides or targeting ligands; these components might act as antigens and provoke antibody responses or unwanted immunostimulation [166]. Studies have noted that certain protein-based nanoparticles or those carrying dietary allergen proteins could pose risks of allergic response in susceptible individuals. Therefore, safety testing for nutraceutical nanocarriers typically includes immunotoxicological assays to detect cytokine release or antibody formation.

Long-term safety and chronic exposure effects are perhaps the most challenging to assess. Nutraceuticals are often taken daily and long-term, so even minimal toxicity per dose can become significant over time. Animal feeding studies (90-day or longer chronic studies in rodents) are usually conducted to look for any signs of organ toxicity, inflammation, or histopathological changes from repeated nanoparticle ingestion [178]. These studies also examine whether nanoparticles interfere with nutrient absorption or gut microbiota composition, as any impact on the microbiome or gut barrier function could have health implications. Interestingly, one consideration with nano-delivery systems is that by enhancing nutrient absorption, they might also nonspecifically enhance the absorption of other compounds. For instance, a nanocarrier that transiently opens tight junctions in the intestine could allow not only the better uptake of a vitamin but also the uptake of unintended substances (e.g., toxins or allergens present in the diet) [177]. This phenomenon necessitates demonstrating that any permeability enhancement is controlled and reversible, without compromising gut integrity. Regulators often require evidence that the nanocarrier does not chronically damage the gut barrier or immune homeostasis.

In summary, nanotoxicological evaluation for biomimetic nutraceutical carriers spans acute cytotoxicity tests, genotoxicity assays, animal studies for ADME (absorption, distribution, metabolism, and excretion) and chronic effects, and immunotoxicity assessments. It is widely recognized that standard toxicity protocols need adaptation for nanomaterials—e.g., ensuring proper particle characterization in test systems and using relevant dose metrics (surface area or particle number in addition to mass) [172]. Agencies like EFSA and FDA are continuously updating testing guidelines as scientific understanding grows. The consistent finding so far is that many nano-delivery systems can be safe if carefully engineered, but each must be scrutinized to avoid scenarios where “nano” properties introduce harm that would not occur with the bulk material.

### 9.5. Safety, Regulatory, and Translational Challenges of Biomimetic Nutraceutical Nanocarriers

The regulatory evaluation of biomimetic nanocarriers in nutraceuticals remains a complex and evolving frontier. A primary difficulty lies in the absence of a universally accepted definition of nanomaterials across jurisdictions. While the European Commission adopts a size-based threshold (<100 nm) as the core criterion for classification [180], the U.S. FDA focuses on functional properties that emerge at the nano-scale, extending consideration to structures up to 1000 nm [169]. This definitional asymmetry generates not only scientific ambiguity but also practical inconsistencies in compliance, as a compound deemed “nano” in one region may not be categorized similarly elsewhere. Rodriguez-Gómez et al. (2025) emphasize this regulatory discord as a key obstacle for industry stakeholders, especially those aiming for multi-jurisdictional commercialization [181].

Beyond definitional concerns, regulatory classification introduces further heterogeneity. The legal identity of a biomimetic nanocarrier—whether it constitutes a novel food, food additive, processing aid, or delivery matrix—varies significantly between countries. In the European Union, any engineered nanomaterial present in the final product is subject to the Novel Food Regulation [182], which entails pre-market safety authorization. In contrast, the U.S. regulatory approach often hinges on whether the nanoformulation modifies the function of the compound or merely its delivery, leading to possible categorization as a dietary supplement or a food additive [183]. This lack of clarity necessitates early regulatory dialog to delineate the applicable pathway, particularly in products operating near the boundary between food and medicine.

From a translational standpoint, regulatory expectations increasingly require nano-specific safety profiles. Preclinical assessment begins with precise physicochemical characterization—size distribution, zeta potential, morphology, and surface chemistry—followed by in vitro assays for genotoxicity, cytotoxicity, and digestibility. Only upon initial safety validation are in vivo studies pursued, often under OECD-compliant protocols [172]. While nutraceuticals typically bypass the stringent efficacy trials mandated for pharmaceuticals, human safety studies, even at a pilot scale, are progressively valued for establishing tolerability, particularly in products with novel carriers or high bioavailability claims.

Despite significant advances in biomimetic nanocarrier design, their regulatory and translational landscape remains underdeveloped compared to that of pharmaceutical nanomedicines. Agencies such as the European Food Safety Authority (EFSA) and the U.S. FDA currently lack specific regulatory frameworks dedicated to nanoformulated nutraceuticals, often applying guidelines designed for either conventional foods or drugs. This regulatory ambiguity limits clear pathways for product classification, safety testing, and health claim substantiation [184].

Moreover, a major barrier to clinical translation is the scarcity of large-scale, controlled human studies validating the efficacy and safety of biomimetic nutraceutical systems. The absence of standardized production protocols, coupled with batch-to-batch variability and complex characterization needs, further complicates regulatory approval. To address these challenges, future efforts must focus on building international consensus for regulatory definitions, developing harmonized testing methods, and promoting interdisciplinary collaboration among regulators, researchers, and industry stakeholders [184].

Another critical component involves manufacturing integrity. The adoption of Good Manufacturing Practice (GMP) standards tailored to nanomaterials is necessary to ensure batch reproducibility, particle homogeneity, and the absence of contaminants such as endotoxins or residual solvents from biological coatings. Moreover, long-term product stability—especially under real-world food matrices—must be documented to rule out physicochemical transformations that could compromise safety. Regulatory bodies may request data on nanoparticle aggregation, leakage of bioactives, or potential degradation byproducts over shelf-life. Such requirements raise the cost and complexity of development but are considered essential for consumer protection.

Post-market surveillance, though historically underdeveloped in the food sector, is now regarded as indispensable for nano-enabled nutraceuticals. The increasing expectation is that manufacturers monitor adverse event reports, collect consumer feedback, and maintain traceability systems, particularly where engineered nanomaterials are involved. Some agencies, such as Health Canada, have begun requiring notification of nano use even in legacy products, suggesting a shift toward retrospective oversight of pre-existing market entries.

Labeling and public disclosure represent an additional axis of regulatory and ethical concern. The European Union’s mandate to include “nano” in the ingredient list of food and supplement products is a notable example of consumer-facing transparency [185]. The ISO guidelines echo this emphasis, promoting voluntary disclosure as a means of fostering informed decision-making and reducing risk perception anxiety [186]. Nonetheless, most other regulatory regions lack equivalent requirements. This creates a dual challenge: avoiding alarmist messaging while ensuring sufficient information is available to consumers, especially those with allergies or other sensitivities potentially exacerbated by nanocarrier components.

Beyond consumer-facing regulations, broader efforts in risk governance are also shaping the policy landscape. The broader policy environment reflects an active effort to modernize risk governance. Initiatives like the Global Summit on Regulatory Science (GSRS) have catalyzed international dialog, promoting the exchange of toxicological data, harmonization of testing standards, and endorsement of safe-by-design principles [187]. In parallel, scientific tools are being adapted to meet the unique demands of nano-scale materials, including organ-on-a-chip models and computational toxicology platforms. These methodologies aim to reduce reliance on animal testing while improving predictive accuracy for human exposure outcomes [174].

Moreover, ethical and environmental dimensions are gaining prominence in policy discourse. The potential for nanoparticulate residues to enter wastewater, agricultural runoff, or soil ecosystems is prompting discussions around life cycle analysis and environmental impact assessments. Furthermore, the intersection between highly efficient nutraceutical nanocarriers and pharmacological thresholds raises critical questions about classification: at what point does a “supplement” become functionally equivalent to a drug? As bioavailability enhancement technologies grow more sophisticated, this legal gray zone is likely to receive closer scrutiny from both food and drug authorities.

In parallel with regulatory modernization, technological tools are being increasingly employed to anticipate nanoparticle behavior and streamline development. Complementing these regulatory and ethical challenges, model-based strategies are increasingly essential in guiding the development and optimization of biomimetic nanoparticle systems [188]. Tools such as physiologically based pharmacokinetic (PBPK) modeling and in silico simulations allow for the prediction of nanoparticle absorption, distribution, and clearance based on physicochemical properties and biological context [189]. These approaches help define optimal particle size, surface charge, and release profiles before extensive in vitro or in vivo testing is performed. Moreover, the integration of machine learning and AI-driven predictive frameworks can enhance formulation screening, reduce trial-and-error cycles, and expand the design space more efficiently [190]. By enabling data-driven decision-making, these model-based strategies significantly improve development throughput while ensuring formulation quality, safety, and performance—key considerations for the translation of biomimetic systems into scalable nutraceutical solutions [191,192].

In this context, the deployment of biomimetic nanocarriers in nutritional science demands a multipronged strategy encompassing definitional standardization, robust safety validation, consumer communication, and ethical oversight. While progress is evident in scientific guidance and policy frameworks, regulatory alignment remains a work in progress. Success will depend on fostering interdisciplinary collaboration among scientists, industry, and policymakers to ensure that innovation is not only accelerated but also safeguarded by a coherent and transparent regulatory ecosystem.

### 9.6. Risk-Based Strategies and Design Space in Biomimetic Nanoparticles

The development and clinical translation of biomimetic nanoparticles require not only innovative design but also systematic risk assessment strategies. A risk-based approach, as recommended by regulatory bodies such as the ICH (International Council for Harmonisation) and GSRS (Global Summit on Regulatory Science), helps define and control the critical quality attributes (CQAs) that influence nanoparticle behavior in vivo—including size distribution, surface charge, encapsulation efficiency, and release kinetics [193].

These parameters significantly affect the disposition of nanocarriers—namely, their absorption, tissue distribution, metabolism, and excretion (ADME). For example, small variations in surface properties may lead to premature clearance or undesired accumulation in off-target organs, increasing toxicity risks or reducing efficacy. Biomimetic particles introduce further complexity due to their hybrid or biological coatings, which may interact unpredictably with the immune system or the microbiome [194].

To address these issues, revisiting the design space of nanoparticle systems is essential. This includes defining acceptable ranges for formulation parameters under Quality by Design (QbD) principles and integrating real-time risk monitoring to adapt formulation or manufacturing processes accordingly. Expanding the design space through adaptive risk control can improve throughput, allow faster optimization of formulations while maintaining safety and performance standards [195].

Furthermore, nanomedicine developers are encouraged to use predictive modeling and simulation tools to anticipate nanoparticle behavior in complex biological systems. By embedding risk-driven thinking early in the development pipeline, it becomes possible to align product design with regulatory expectations and clinical needs, accelerating the translation of biomimetic nutraceutical technologies into safe, effective, and scalable interventions.

## 10. Applications in Personalized Nutritional Therapy

Personalized nutritional therapy represents a rapidly advancing domain focused on customizing dietary interventions according to individual characteristics such as genetic background, gut microbiota profile, metabolic status, and lifestyle habits [196,197]. The primary objective of this approach is to optimize health outcomes by aligning nutritional strategies with the specific physiological demands of each person. Within this, biomimetic delivery systems provide innovative solutions by enabling the accurate, adaptive, and targeted administration of nutraceuticals tailored to personal needs. These technologies hold the potential to transform the landscape of personalized nutrition through enhanced therapeutic efficiency, reduced adverse effects, and the integration of real-time dietary modulation.

Biomimetic nanocarriers, such as lipid-based vesicles, exosomes, and polymeric nanoparticles, have shown great promise in delivering bioactives in a controlled and individualized manner [198]. For instance, smart nano-delivery platforms that respond to internal triggers, such as pH, temperature, or enzyme concentration, can ensure that nutraceuticals are released at the right time and location in the body [199]. This is particularly valuable in managing metabolic diseases, gastrointestinal conditions, and nutrient deficiencies where conventional supplementation lacks specificity. Recent developments, such as those shown by Li et al. regarding the use of cell membrane-coated nanoparticles and microenvironment-responsive hydrogels, have further enhanced the precision and efficiency of nutrient delivery systems in personalized health applications [200,201]. These innovations pave the way for the integration of nutraceuticals into precision medicine frameworks, supporting preventive and therapeutic interventions tailored to individual needs.

Moreover, advances in biosensor technologies and wearable health-monitoring devices are enabling the integration of biomimetic nutraceuticals into real-time, feedback-driven nutritional strategies [202,203]. These systems allow for the continuous tracking of physiological parameters—such as glucose levels, hydration status, or metabolic biomarkers—and facilitate the dynamic adjustment of nutrient delivery according to the individual’s current biological state [204]. Such personalization enhances the therapeutic effectiveness of nutraceuticals and supports proactive, preventive healthcare interventions [205]. As these technologies continue to evolve, they are expected to play a pivotal role in the development of closed-loop, adaptive nutrition platforms that combine diagnostics, smart delivery, and personalized intervention in a unified system.

Recent advancements in artificial intelligence (AI) and biomimetic technologies are revolutionizing personalized nutrition and health management. Kumar et al. discuss AI-driven personalized nutrition applications that monitor dietary intake, physical activity, and health parameters, providing tailored dietary recommendations to enhance overall wellness [206,207]. Complementing this, Beltrán-Velasco and Clemente-Suárez highlight the gut microbiota’s role as a model for biomimetic innovations, emphasizing its influence on immunity, metabolism, and the gut–brain axis, which can inspire the development of personalized probiotics and bio-inspired biosensors [207]. Furthermore, Han et al. explore biomimetic nano-drug delivery systems, underscoring their potential to optimize therapeutic efficacy through controlled and targeted release mechanisms, thereby enhancing treatment outcomes [208].

Additionally, recent advancements in 3D printing technologies and biomimetic materials are significantly enhancing the field of personalized nutrition and healthcare. Singh et al. (2024) discuss how 3D printing enables the precise fabrication of customized dosage forms, offering controlled release patterns and patient-specific medications, thereby improving therapeutic outcomes in drug delivery and regenerative medicine [209]. Additionally, Paul-Chima et al. emphasize the importance of optimizing absorption, distribution, metabolism, and excretion (ADME) dynamics in nutraceutical delivery systems to enhance therapeutic potency and clinical impact, highlighting the integration of various delivery strategies to achieve this goal [210]. Furthermore, a recent study introduced an approach to prepare fully artificial extracellular vesicle (EV) biomimetics, maintaining the natural lipidic composition, which could revolutionize targeted therapy and diagnostics [211].

Collectively, these studies underscore the transformative potential of integrating 3D printing, AI, and biomimetic strategies in developing personalized nutritional therapies and advanced health interventions.

## 11. Practical Applications

Personalized nutrition is gaining prominence as a discipline that seeks to tailor dietary interventions according to the individual genetic, metabolic, and microbiological characteristics of each person. With the increasing awareness of the importance of precise and adequate nutrition, novel strategies have been developed to enhance the delivery of nutraceuticals, thereby improving health outcomes. Among the technologies in this field, biomimetic strategies are emerging, primarily through the use of nanomaterial-based nutraceutical delivery systems. Some of the practical applications developed in this study include biomimetic strategies in nutraceutical delivery systems, advances in nanomedicine and bionanotechnology for nutritional health, and the integration with real-time health-monitoring technologies.

Biomimetic strategies in nutraceutical delivery systems: Biomimetic strategies aim to replicate natural biological processes to enhance the efficacy of treatments. In the context of nutraceutical delivery, this involves the use of delivery systems that mimic the biological functions of cellular and molecular systems. Nanobiomimetic vehicles, such as lipid nanoparticles, exosomes, and microenvironment-responsive hydrogels, have shown significant potential for improving the administration of bioactive compounds. For instance, lipid-based nanoparticles, which mimic the structure of cell membranes, enable controlled and targeted release of nutraceuticals. Similarly, smart delivery platforms that respond to internal triggers, such as pH, temperature, or enzyme concentration, ensure that nutraceuticals are released at the appropriate time and location within the body, thereby optimizing therapeutic outcomes.

Advances in nanomedicine and bionanotechnology for nutritional health: Bionanomedicine has emerged as a key field for enhancing nutraceutical delivery. As technology advances, bionanomedicine solutions are enabling the design of increasingly sophisticated delivery systems that can be adapted to the individual needs of patients. The integration of nanotechnology into personalized nutrition has a significant impact, particularly in the management of metabolic diseases, nutritional deficiencies, and gastrointestinal disorders. Recently, nanoparticles coated with cell membranes have been developed, which can mimic the characteristics of human cells and improve the biocompatibility of treatments. These innovations have been essential in ensuring the efficacy of nutraceuticals in the treatment of chronic diseases and in the modulation of the gut microbiota, one of the most studied areas in personalized nutrition. The use of controlled release systems has also proven beneficial in improving the bioavailability of vitamins, minerals, and other bioactive compounds. The precise administration of these compounds allows nutraceuticals to be released continuously and in appropriate doses according to the body’s needs, which is particularly important for the treatment of nutritional deficiencies and the management of metabolic diseases such as diabetes and obesity.

Integration with real-time health-monitoring technologies: The integration of biomimetic systems in personalized nutrition is enhanced using real-time health-monitoring technologies. Wearable devices and biosensors are enabling the continuous collection of physiological parameters, such as glucose levels, hydration, and metabolic biomarkers. This information is crucial for making dynamic adjustments in nutraceutical delivery, allowing for even more precise personalization of nutritional therapy.

## 12. Challenges and Future Perspectives

Biomimetic strategies for nutraceutical delivery have emerged as a highly promising innovation in personalized medicine and advanced nutrition. The application of nanotechnology and biomimetic systems to optimize the delivery of bioactive compounds has demonstrated significant efficacy in enhancing nutrient bioavailability, reducing adverse effects, and tailoring treatments to individual needs. However, despite substantial progress in this field, considerable challenges remain that must be addressed to maximize the impact of these technologies on nutritional health and ensure their widespread adoption in clinical practice.

Despite the promising advancements, several challenges must be overcome for biomimetic strategies to become a viable and accessible option in the realm of nutritional health. One of the primary obstacles is the complexity and cost of the technologies involved. The development of advanced nanoparticles, exosomes, and smart delivery systems necessitates considerable investment in research and development, which could limit the accessibility of these solutions for many patients. Moreover, the large-scale manufacturing of these personalized delivery systems still faces technological barriers that need to be resolved to ensure the efficient and cost-effective production of these products.

Another significant challenge is the regulation of nanotechnology-based nutraceuticals. While nutraceuticals themselves are already regulated in many countries, the novel biomimetic technologies used for their delivery may not be adequately covered by existing regulations. Regulatory authorities must update and adapt their policies to ensure that these products are safe and effective. This involves establishing clear guidelines on the safety assessment of nanoparticles, exosomes, and other biomimetic systems, as well as the authorization of new delivery methods. Safety is also a central concern. Although biomimetic systems have the potential to reduce side effects and improve the precision of nutraceutical delivery, they may also pose unknown risks, particularly in terms of long-term biocompatibility. Toxicity studies and clinical trials are essential to ensure that these systems do not produce unexpected adverse effects or unwanted immunological reactions. Additionally, the use of technologies such as exosomes, which mimic human body cells, raises questions about the possibility of interaction with the immune system and their long-term impact on health.

One of the most promising aspects of biomimetic strategies is their integration with real-time monitoring technologies, such as biosensors and wearable health-monitoring devices. These devices enable the continuous collection of physiological data, which facilitates the adaptation of nutritional treatments to the changing needs of each individual. As new biosensors and monitoring technologies are developed, it will become possible to dynamically adjust the release of nutraceuticals based on parameters such as glucose levels, hydration, and metabolic biomarkers. However, the integration of these technologies into clinical practice presents additional challenges. The collection of large amounts of personal data raises concerns about privacy and information security. Real-time monitoring systems must be protected by strict protocols to ensure that patient data is handled ethically and securely. Furthermore, healthcare professionals will need to be trained to interpret and utilize this data correctly to make informed decisions about nutraceutical administration.

The perspectives for the utilization of biomimetic strategies in nutraceutical delivery are exceptionally encouraging. As science and technology continue to advance, biomimetic-based delivery systems are anticipated to offer increasingly personalized, effective, and safe solutions. A particularly exciting development is the integration of artificial intelligence (AI) with biomimetic systems, which will enable an unprecedented level of customization in nutraceutical administration. For instance, AI algorithms could analyze real-time data on an individual’s physiological status, such as glucose levels, gut microbiota composition, or metabolic biomarkers, to dynamically adjust the release of nutraceuticals according to individual requirements. This continuous and personalized feedback would enhance the efficacy of treatments and facilitate more precise preventative medicine.

Furthermore, the application of emerging technologies like 3D printing is revolutionizing the creation of customized dosage forms. By printing controlled-release devices tailored to the biological characteristics of each patient, it will be possible to offer highly specific treatments that maximize efficacy and convenience. These types of innovations have the potential to improve not only nutritional health but also treatments for metabolic diseases, nutritional deficiencies, and gastrointestinal disorders.

## 13. Conclusions

Biomimetic delivery systems represent a transformative approach in nutraceutical science, offering innovative solutions to long-standing challenges of bioavailability, stability, and targeted efficacy. This review has synthesized recent advances in lipid-based carriers, cell membrane-coated nanoparticles, exosomes, and bio-inspired nanomaterials, all of which demonstrate significant potential to enhance the therapeutic impact of nutraceuticals. By emulating biological mechanisms, these systems improve absorption, protect sensitive compounds, and enable site-specific delivery, paving the way for more effective strategies in managing oxidative stress and chronic diseases. While translational hurdles remain, including safety validation and regulatory standardization, the integration of biomimetic technologies marks a critical step toward precision nutrition and personalized health interventions. Continued interdisciplinary research will be key to unlocking the full clinical promise of these next-generation nutraceutical platforms.

## Figures and Tables

**Table 1:** Key terminology in biomimetic and nano-enabled nutraceuticals.

Term	Definition	Example/Context
Bionanomedicine	The application of nanotechnology in biology and medicine, particularly for therapeutic purposes.	Nano-drug carriers for cancer or metabolic diseases.
Biogenic	Nanoparticles synthesized using biological entities (e.g., plants and microbes) without synthetic chemicals.	Silver nanoparticles from plant extracts.
Bio-inspired	Designs or materials that take inspiration from biological systems but may not replicate them exactly.	Self-cleaning surfaces mimicking lotus leaves.
Biomimetic	Materials or systems that replicate biological structures or functions closely.	Cell-membrane-coated nanoparticles for immune evasion.
Nanozymes	Nanomaterials with intrinsic enzyme-like catalytic activity.	Iron oxide nanoparticles acting as peroxidase mimics in detoxification.

**Table 2:** Summary of Biomimetic Strategies in Nutraceutical Delivery.

Task	Challenge	Importance of Using Biomimetic Property	Perceived Nano Benefit	Nanotechnology Used	Study
Delivery of lipophilic nutraceutical	Protect from hydrolysis and enzymatic degradation under GI conditions	Oil-in-water nano-emulsions can dissolve large amounts of lipophilic compounds	Improved solubility and protection in the GI tract	Lipid nanoparticles	[64]
Targeted delivery of antioxidants	Limited cellular uptake and poor tissue targeting	Mimic natural membrane interactions for targeted delivery	Enhanced cellular uptake and antioxidant efficacy	Cell membrane-coated nanoparticles	[14,74]
Protection and delivery of polyphenols	Instability during digestion	Encapsulation in protein-based nanocarriers	Improved gastric resistance and sustained release	Casein micelles and gelatin nanoparticles	[36,41]
Enhancing antioxidant delivery to neural tissue	Crossing biological barriers like the BBB	Use of cell-like vesicles with targeting ligands	Targeted delivery and improved retention	Selenium nanoparticles and exosome-like vesicles	[12,78]
Anti-inflammatory delivery in IBD	Need for colon-specific delivery	Mucoadhesive and pH-sensitive carriers	Selective release in the colon; reduced systemic side effects	Chitosan/alginate nanoparticles	[44]
Enhanced delivery of curcumin	Low bioavailability and rapid metabolism	Phospholipid-based or hybrid carriers	3- to 9-fold increase in absorption	Liposomes, polymeric micelles, and hybrid nanoparticles	[66,79]

**Table 3:** Challenges in transitioning from synthetic to biomimetic nanocarriers for nutraceutical applications.

Nanoparticle Technology	Perceived Nano Benefit	Challenge for Biomimetic Strategy	Current Biomimetic Strategy	Limitations
Synthetic polymeric nanoparticles	Controlled release, stability, and scalable production	Low biocompatibility and immune recognition	Cell membrane-coated nanoparticles (e.g., RBC and macrophage membranes)	Complex fabrication, source standardization, and potential immune response
Liposomes	Good solubility for lipophilic compounds and biocompatibility	Instability during storage and rapid clearance in vivo	Stealth liposomes (PEGylated or membrane-coated)	PEG-related immune responses and limited long-term stability
Nanoemulsions	Enhanced absorption and GI stability for lipophilic nutraceuticals	Droplet size and surfactant toxicity concerns	Food-grade oil-in-water nanoemulsions using natural emulsifiers	Emulsion breakdown and sensitivity to digestive enzymes
Metal/metal oxide nanoparticles	High antioxidant and antimicrobial activity	Potential toxicity and accumulation in tissues	Biogenic synthesis using plant extracts or microbes	Batch variability, low scalability, and regulatory ambiguity
Protein-based nanoparticles	Digestive responsiveness and natural compatibility	Reproducibility and environmental sensitivity	Zein-, casein-, and gelatin-based nanoparticles	Sensitivity to pH, enzymatic degradation, and storage issues

## Data Availability

Not applicable.
